# Advances in Electrophysiological Research

**DOI:** 10.35946/arcr.v37.1.05

**Published:** 2015

**Authors:** Chella Kamarajan, Bernice Porjesz

**Affiliations:** Chella Kamarajan, Ph.D., is assistant professor of psychiatry and behavioral sciences; Bernice Porjesz, Ph.D., is professor of psychiatry and behavioral sciences and director of the Henri Begleiter Neurodynamics Laboratory, SUNY Downstate Medical Center, Brooklyn, New York.

**Keywords:** Alcoholism, alcohol use disorder, alcohol-impaired offspring, risk factors, family risk factors, brain function, pathophysiological processes, electrophysiological research, electrophysiological measures, electroencephalogram, event-related potentials, event-related oscillations, genes, genetic factors

## Abstract

Electrophysiological measures of brain function are effective tools to understand neurocognitive phenomena and sensitive indicators of pathophysiological processes associated with various clinical conditions, including alcoholism. Individuals with alcohol use disorder (AUD) and their high-risk offspring have consistently shown dysfunction in several electrophysiological measures in resting state (i.e., electroencephalogram) and during cognitive tasks (i.e., event-related potentials and event-related oscillations). Researchers have recently developed sophisticated signal-processing techniques to characterize different aspects of brain dynamics, which can aid in identifying the neural mechanisms underlying alcoholism and other related complex disorders. These quantitative measures of brain function also have been successfully used as endophenotypes to identify and help understand genes associated with AUD and related disorders. Translational research also is examining how brain electrophysiological measures potentially can be applied to diagnosis, prevention, and treatment.

The discovery and recording of electrical activity (electroencephalography [EEG]) in the human brain in 1924 by the German physician Hans Berger (Collura 1993; Haas 2003) has led to numerous scientific breakthroughs and clinical applications (Borck 2005; Gloor 1994). Recording brain activity in humans using scalp electrodes provides a noninvasive, sensitive measure of ongoing brain function during resting state and during sensory and cognitive tasks (Porjesz et al. 2005). In contrast to neuroimaging methods, such as functional magnetic resonance imaging (fMRI), which have poor temporal resolution limited by the biophysics of the hemodynamic response, these electrophysiological methods have temporal resolution in the millisecond range and reflect the dynamic balance between excitation and inhibition in brain neural networks. Although fMRI methods are known to have superior spatial resolution, the data processing of neuroimaging methods (e.g., fMRI and positron emission tomography [PET]) frequently diminishes this acclaimed spatial resolution (especially during postprocessing of the data, which often involves “spatial smoothing” and/or averaging across voxels within a region of interest). In contrast, modern EEG recorded with up to 256 channels considerably improves its spatial resolution. Other comparative advantages of electrophysiological methods include (1) superior test–retest reliability of EEG within subjects and across labs, (2) relative ease of use, (3) lower cost, (4) applicability in larger studies to answer more complex and interesting questions than can not be answered with PET or fMRI, and (5) validity of EEG measures as a direct neural correlate (i.e., the EEG does not rely on an assumption about neurovascular coupling).

To date, these electrophysiological measures of brain function remain the most valuable method to study the sensory, motor, and cognitive phenomena as they unfold in the human nervous system. Scalp electrical activity results from ensembles of neurons firing in synchrony, which produce oscillatory activity. The oscillatory patterns, which have specific frequency-band characteristics, facilitate neural communication in the brain. The electrophysiological characteristics of individuals are affected by genes that control or modulate a variety of neurotransmitters and other biological factors. Electrophysiological methods have unique and far-reaching applications ranging from clinical and cognitive neuroscience to gene identification and can inform the field regarding prevention and neuropharmacological intervention in a variety of neuropsychiatric conditions, especially substance use disorders (SUDs) (Ho et al. 2010; Schuckit 2000). Further, research has firmly established the utility of electrophysiological methods in many aspects of alcoholism (for recent reviews, see Campanella et al. 2009; Pandey et al. 2012*a*; Porjesz and Rangaswamy 2007; Porjesz et al. 2005; Rangaswamy and Porjesz 2008*a*,*b*, 2014).

Alcoholism is a neuropsychiatric disorder with complex etiological contributions from genetic and environmental factors and their interactions (Kendler et al. 2003). Electrophysiological measures have served as effective “endophenotypes”—intermediary measures of neuropsychiatric function that are correlated with alcoholism and are involved in the pathway between genotype and alcoholism (Porjesz et al. 2005). Electrophysiological measures of brain function are highly heritable, and strong evidence suggests that some electrophysiological characteristics observed in alcoholics already are present in their offspring prior to exposure to alcohol or drugs, thus preceding the development of alcoholism. These electrophysiological endophenotypes may serve as valuable biomarkers for the genetic vulnerability underlying alcoholism (for reviews, see Begleiter and Porjesz 2006; Porjesz et al. 2005).

Electrophysiological activity can be recorded as continuous EEG during the resting state, reflecting ongoing mental states (Niedermeyer and Lopes da Silva 2005), or as time-locked event-related brain activity during cognitive tasks. The latter can be analyzed in the time domain as event-related brain potentials (ERPs), representing neural processing during a variety of sensory and cognitive tasks (Rugg and Coles 1996), or with newer time–frequency analyses, yielding event-related oscillations (EROs), or time- and frequency-specific oscillatory patterns during neurocognitive tasks (Basar 1999*a*,*b*). This article highlights recent research using EEG, ERP, and ERO methods recorded during wakeful or active states in alcoholics and in offspring of alcoholics from densely affected families (i.e., with multiple alcohol-dependent relatives), who are considered to be high-risk (HR); it summarizes the most useful and sophisticated techniques that are available for alcoholism research, and reviews advances in signal processing tools and techniques. Although acute effects of alcohol are not discussed for each of the techniques in this review (for a review, see Rangaswamy and Porjesz 2014), these studies are briefly mentioned with respect to a few of the advanced techniques that do not otherwise have any studies on alcoholics or HR subjects, in order to demonstrate some alcohol-related applications. For each method, the article also examines major findings in alcoholism and possible translational applications of these electrophysiological measures to diagnosis, prevention, treatment, and rehabilitation, including the utility of these measures as highly heritable and sensitive endophenotypic markers for gene identification, with potential for possible drug development for alcoholism.

## Resting/Spontaneous EEG: Findings and Prospects

EEG records the spontaneous, continuous neural activity during various mental states and under a variety of conditions, such as eyes-closed relaxed state, eyes-open steady state, meditation, hypnosis, various stages of sleep, coma, and other normal/altered states of consciousness (Niedermeyer and Lopes da Silva 2005). EEG records a complex signal that can be decomposed into a wide range of frequencies using the Fast Fourier Transform (FFT) technique (Cooley and Tukey 1965), based on the principle that any time series can be represented as a summation of sine waves of different frequencies, each with its own phase and amplitudes (Boashash 1992). This section outlines the use of waking resting EEG power and coherence measures in alcoholics and HR offspring and discusses other novel signal processing methods using resting EEG data.

## EEG Power in Alcoholism

### Low-Frequency [Delta (1 to 4 Hz) and Theta (4 to 7 Hz)] Activity

Similar to phylogenic development characterized by awake delta state in reptiles and theta and alpha states in mammals (Knyazev 2012), awake EEG activity in human infancy is dominated by low-frequency delta rhythm during the first 2 years of life followed by a transition toward a gradual decrease in slow delta and theta activity as well as a gradual increase in faster alpha and beta bands almost linearly across development from childhood through adolescence to adulthood (e.g., John et al. 1980). However, increased delta activity in awake human adolescence and adulthood has been related to many neurological disorders as well as several psychiatric conditions, such as schizophrenia (Begic et al. 2000; Karson et al. 1987; Sponheim et al. 2000). In alcoholism, early EEG studies reported that abstinent alcoholics showed increased delta power (Begleiter and Platz 1972; Kaplan et al. 1985; Volavka et al. 1985). In contrast, studies have found decreased slow-wave activity in alcoholic patients in the delta band (Saletu-Zyhlarz et al. 2004) as well as in both delta and theta bands (Coutin-Churchman and Moreno 2008; Coutin-Churchman et al. 2003, 2006). Additional research among people with SUDs has reported similar findings of decreased slow-wave activity in the delta band (Alper et al. 1998), as well as in both delta and theta bands (Prichep et al. 1996). In a study of binge drinkers, Courtney and Polich (2010) reported that high–binge drinkers exhibited more spectral power in the delta (0 to 4 Hz) and fast-beta (20 to 35 Hz) bands than non–and low–binge drinkers. Taken together, findings on delta power in alcoholism seem to be inconclusive.

Human resting theta rhythm has its maximum power in the posterior scalp region; the normal adult waking EEG record contains a relatively lower amount of theta power compared with other fast frequencies (cf. Porjesz et al. 2005). Studies have reported that alcoholic subjects manifest increased resting theta power (Fein and Allen 2005; Propping et al. 1981, 1992; Rangaswamy et al. 2003), although some studies by Coutin-Churchman and colleagues (2003, 2006) have reported decreased theta activity in alcoholics. It is also interesting to note that HR offspring of alcoholics from densely affected families do not manifest the abnormal theta power seen in alcoholics, in contrast to several other EEG measures. Hence these theta abnormalities in alcoholics are likely the result of chronic alcohol intake on brain function (Rangaswamy and Porjesz 2014). As reviewed below (“Electrophysiological Measures as Endophenotypes for Alcoholism”), genetic research has found linkage and association of a cholinergic muscarinic neurotransmitter receptor gene (*CHRM2*) with two theta oscillation measures: (1) theta ERO during the processing of target stimuli during an oddball task 1 and (2) resting eyes-closed EEG high-theta (6 to 7 Hz) interhemispheric coherence (Jones et al. 2004, 2006*a*; Porjesz and Rangaswamy 2007; Rangaswamy and Porjesz 2008*b*).

### Alpha Band (8 to 12 Hz)

The alpha rhythm is predominant when an individual is awake and relaxed, and has its maximum power in the eyes-closed condition over the occipital regions. Human alpha oscillations (during resting state as well as during cognitive processing) are related to higher cognitive function and brain maturation. Alpha activity in children starts only after 3 years of age, almost parallel to the development of speech, and the posterior dominant alpha rhythm continues to develop until the age of 16 (cf. Basar 2012). Many early EEG studies showed that alcoholics manifest less prevalent and lower alpha power compared with control subjects (for reviews, see Begleiter and Platz 1972; Propping et al. 1981). However, some studies failed to replicate this finding of low resting alpha power in alcoholics (Enoch et al. 1999; Fein and Allen 2005; Pollock et al. 1992). Researchers found that a decrease in slow alpha activity in alcoholics is more pronounced in relapsers than in those who maintain abstinence (Saletu-Zyhlarz et al. 2004). Further, gender- as well as ethnicity-related alpha findings have been reported in offspring of alcoholics (Ehlers and Phillips 2003; Ehlers and Schuckit 1991; Ehlers et al. 1996; Finn and Justus 1999). Manifestations of the low-voltage alpha (LVA) variants may be influenced by ethnicity and gender, whereas the findings on alpha power are equivocal. The association of LVA variants in females to a catechol-o-methyltransferase (COMT) gene and to anxiety and alcoholism is discussed in a later section (see “Electrophysiological Measures as Endophenotypes for Alcoholism”).

### Beta Band (12 to 28 Hz)

Beta-frequency rhythms in resting EEG are prevalent in the awake and alert state. Several studies have reported increased beta power in the resting EEG of alcoholics (Bauer 2001; Costa and Bauer 1997; Fein and Allen 2005; Propping et al. 1981; Rangaswamy et al. 2002; Winterer et al. 1998). Increased beta activity often is taken as a sign of increased neural excitability (hyperexcitability or central nervous system [CNS] disinhibition); it is apparent in alcoholics (Porjesz et al. 2005), where it has been shown to be a predictor of relapse (Bauer 2001; Saletu-Zyhlarz et al. 2004), and has been reported in HR relatives of alcoholics, including both male and female offspring (Finn and Justus 1999; Gabrielli et al. 1982; Pollock et al. 1995; Rangaswamy et al. 2004*b*), although it is more robust in males (Finn and Justus 1999; Gabrielli et al. 1982; Pollock et al. 1995; Rangaswamy et al. 2002, 2004*b*). This suggests that the neural hyperexcitability observed in alcoholics may antecede the development of alcoholism (Porjesz et al. 2005). The association of beta and neural hyperexcitability to a gamma-aminobutyric acid type A (GABA_A_) receptor gene (*GABRA2*) is discussed below (see “Electrophysiological Measures as Endophenotypes for Alcoholism”).

## EEG Methods and Advances

### Dipole Source Modeling for EEG Data (FFT Dipole Approximation)

Following the rapid growth of quantitative EEG (qEEG) and digital signal processing in the late 1960s, several methods to track neural generators of EEG and ERPs were introduced (see the section “Dipole Source Modeling for ERP Data”). Dipole source modeling is one of the early techniques attempting to solve the inverse problem of deriving the source configuration from recorded scalp potentials, by using mathematical simulations and modeling to understand the spatiotemporal complexity of both ongoing and evoked electrical scalp activity (Lehmann and Michel 1989; Scherg 1990; Scherg and Berg 1996; Scherg and Picton 1991) (for a detailed account on source localization methods, see Pizzagalli 2007). The cerebral sources of EEG/MEG data are estimated using mathematical modeling approaches. Specifically, for EEG data, researchers introduced a prominent method known as the FFT dipole approximation model (FFT-DA) (Lehmann and Michel 1989; Michel et al. 1992). The FFT-DA method enabled the computation of intracerebral, three-dimensional location of single dipole sources by modeling multichannel EEG data in the frequency domain using a potential distribution map containing polarity and phase information. This approach has been used predominantly to compute intracerebral sources of various EEG frequency bands in clinical conditions, such as schizophrenia (Dierks et al. 1995), depression (Dierks et al. 1993), Alzheimer’s disease (Huang et al. 2000), and epilepsy (Ebersole 1991; Verhellen and Boon 2007). Although there are no studies on EEG dipole modeling in alcoholism, it may be worth revisiting this method, as dipole modeling in EP/ERP data has been successfully applied to alcoholics (Hegerl et al. 1995). Dipole modeling algorithms have been often criticized as making unrealistic assumptions about the number of likely generators and their size or orientation (Bauer 2001). Further, when the assumption of a single oscillating dipole generator is unwarranted or unlikely, resulting source identification may be less reliable (Pizzagalli 2007).

### Resting EEG Coherence

Coherence is a measure of “coupling” or functional association between two brain regions (Nunez 1981, 1995). Coherence between distant brain regions is related to higher-order cognitive function, is specific to mammalian and human brains, and does not occur in the neural networks of invertebrates and other lower animals (Basar and Guntekin 2009; Bullock and Basar 1988). Measuring coherence with the objective of discovering groups of neurons that act together in a coherent fashion (i.e., Hebbian cell assemblies) (Hebb 1949), has a long history (Horwitz 2003). EEG coherence reflects the dynamic functional interrelation between spatially separated electrode sites (Horwitz 2003). Coherence is computed as a normalized coefficient of cross-spectral power between two signals, and it estimates the consistency of phase, weighted by amplitude, between any pair of signals for each frequency (cf. Srinivasan et al. 2007). As a noninvasive method at the macroscopic level, EEG was the first method to examine the functional connectivity between different cortical regions, by correlating different features of the spatiotemporal waveforms associated with measured electrical activity using several techniques (Adey et al. 1961; Barlow and Brazier 1954; Gevins et al. 1985; Livanov 1977; Pfurtscheller and Andrew 1999). For instance, Gevins and colleagues (1985) measured dynamically changing cross-correlation of the time series between a pair of electrodes; Pfurtscheller and Andrew (1999) computed the correlation in the frequency domain between EEG signals at different scalp sites. Chorlian and colleagues (2009) reported frequency-specific topographical patterns in bipolar EEG coherence (which is devoid of volume conduction effects), and found an interesting similarity of these patterns with those obtained by resting state networks identified by fMRI studies.

EEG coherence has been used to examine cognitive and emotional processes (Kislova and Rusalova 2009; Marosi et al. 1999; Martin-Loeches et al. 2001; Thatcher et al. 2005), cognitive impairment (Babiloni et al. 2010; Gasser et al. 2003; Marosi et al. 1997), and various clinical conditions (Barry et al. 2005; De Vico Fallani et al. 2010; John 2009; Kumar et al. 2009; Shaw et al. 1983). It also has been used to index brain maturation (Barry et al. 2004; Gasser et al. 2003; Hanlon et al. 2005; Thatcher 1992, 1998; Thatcher et al. 1987, 2008). Gender differences in coherence also have been observed (Hanlon et al. 1999; Koles et al. 2010).

Increased EEG coherence in slower frequencies (e.g., delta band) and decreased coherence in higher frequencies (e.g., high alpha and beta bands) have been reported in alcoholics (Kaplan et al. 1985; Tcheslavski and Gonen 2012). Michael and colleagues (1993) found increased delta coherence (F3 to F4); increased fast-beta coherence (F3 to F4 and C3 to C4); and also reported an increase in theta, alpha, and slow-beta coherence at a central electrode pair (C3 to C4). They also found that alcohol-naïve first-degree relatives of alcoholics had shown significantly higher alpha and beta coherence than alcoholics (frontal and parietal regions) and healthy control subjects (frontal and centroparietal regions). However, there are no follow-up studies to confirm these findings in HR subjects. Winterer and colleagues (2003) reported that bilateral, intrahemispheric, posterior coherences were significantly increased in the alpha and beta bands in both long-term abstinent and nonabstinent alcohol-dependent study participants, particularly when depressiveness was included as a covariate. Abstinent alcoholics also have been reported to manifest increased resting interhemispheric high theta (6 to 7 Hz) coherence with a more posterior topography than control subjects (Porjesz and Rangaswamy 2007; Rangaswamy and Porjesz 2008b). This high-theta coherence phenotype was found to be associated with both a GABA_A_ (*GABRA2*) and a cholinergic receptor (*CHRM2*) gene (see “Electrophysiological Measures as Endophenotypes for Alcoholism”).

### Graph Theoretical Methods

From a graph theoretical perspective, the brain is conceptualized as a networked system composed of regions (nodes) functionally connected with different brain regions by “paths,” which are weighted by measures of statistical dependencies between their electrical activity at the nodes they connect (De Vico Fallani et al. 2012). Some properties of interest are whether there are groups of nodes more strongly connected to other nodes in their group than to nodes in other groups, whether there are paths of high connectivity between most nodes, and whether some nodes (hubs) have many paths of high connectivity with other nodes. Graph theoretical analysis offers a powerful way to understand the topological principles of brain networks in normal and clinical populations and across development (He and Evans 2010). These methods have been applied to resting state as well as task-related data (Reijneveld et al. 2007). Magnetoencephalographic (MEG) studies also have used graph theoretical methods. Although EEG and MEG signals originate from the same neurophysiological processes, magnetic fields recorded using scalp sensors are less distorted than electrical potentials by the skull and scalp. However, MEG detects only tangential, intracellular currents, whereas EEG is related to both radial and tangential extracellular currents emanating from cortical sulci and gyri (Babiloni et al. 2009; Cohen and Cuffin 1983). Using the graph theoretical methods with EEG/MEG data, researchers have analyzed brain networks in several clinical conditions, including schizophrenia (Bassett et al. 2009; Rubinov et al. 2009), epilepsy (van Dellen et al. 2009), depression (Leistedt et al. 2009), and bipolar disorder (Kim et al. 2013). In an MEG study using graph theoretical approaches, Stam and colleagues (2009) showed that patients with Alzheimer’s disease had decreased connectivity of hubs in their brain networks and that highly connected neural network hubs were especially at risk in this disease. This result also was compatible with a previous fMRI-based brain network study in Alzheimer’s disease (Supekar et al. 2008). In alcoholism, Sakkalis and Marias (2012) elicited statistically significant graph-theoretic indices that quantified cognitive processes in the EEG data of alcoholic subjects. Sakkalis and colleagues (2014) found that alcoholics had impaired (graph theory based) synchronization and loss of lateralization during the rehearsal process, most prominently in alpha (8 to 12 Hz) and beta (13 to 30 Hz) bands, compared with control subjects. Further studies are under way in alcoholics and HR offspring.

### Microstate Analysis

The topographic distributions of the EEG scalp potential during resting state does not change randomly or continuously over time but remains stable over periods of about 100 to 200 ms; these quasi-stable topographic distributions of the electrical field potential have been termed “microstates” (Lehmann et al. 1998) and are considered to be “atoms of thought” (Lehmann et al. 2004). It has been proposed that there is an intrinsic connection between the fast neuronal activity and slow hemodynamic fluctuations as revealed by concurrent EEG and blood oxygenation level–dependent (BOLD)-fMRI studies (Britz et al. 2010; Musso et al. 2010). Therefore, sequences of EEG microstates are assumed to be electrophysiological signatures of resting state networks of the BOLD signals (Yuan et al. 2012). Microstate analysis yields a repertoire of short-lasting functional states, termed “classes,” described by topographic pattern, occurrence frequency, duration, and temporal sequence, or “syntax” (Koenig et al. 2002; Schlegel et al. 2012). These variables showed a lawful, complex evolution with advancing age from early childhood to late adulthood (Koenig et al. 2002).

Microstate analyses have been implemented in several clinical conditions, including schizophrenia (Lehmann et al. 2005; Nishida et al. 2013; Strelets et al. 2003), depression (Strik et al. 1995), attention deficits in children (Brandeis et al. 1998; Latchoumane et al. 2013), and panic disorder (Kikuchi et al. 2011; Wiedemann et al. 1998). Although no studies have been done to date, microstate analyses may be potentially useful in alcoholism as well.

## ERPs: Findings and Prospects

ERPs are time-locked voltage fluctuations of the scalp-recorded neuroelectric activity in response to a sensory, motor, or cognitive event, extracted by signal processing methods such as filtering and trial averaging (Picton et al. 2000). These electrical potentials are obtained by averaging single trial EEG epochs time locked to a stimulus or event and represent large numbers of neural elements acting in synchrony during information processing, from early sensory perception to higher cognitive processing. Early components (less than 100 ms) index sensory reception, whereas the later components (more than 100 ms) index higher cognitive processing, such as selective attention, memory updating, semantic comprehension, and other cognitive activity (Duncan et al. 2009). ERP components are identified and interpreted based on their eliciting conditions, polarity (positivity or negativity), timing (latency), and scalp distribution or topography (cf. Kamarajan and Porjesz 2012). The latency (time of occurrence of an ERP phenomenon in milliseconds) reflects neural processing time, whereas the amplitude (magnitude of an ERP component in microvolts) has been related to the neural resources available to process a stimulus or event (Rugg and Coles 1996). Frequently studied ERP components include P1, N1, P2, N2, P3 (P300), N4 (N400), mismatch negativity (MMN), contingent negative variation (CNV), and bereitschaftspotential (BP) or readiness potential. These components are obtained using specific ERP tasks, such as oddball tasks, Go/No-Go tasks,^2^ continuous performance task (CPT),^3^ stop-signal tasks,^4^ monetary gambling tasks (MGT), decision-making tasks, memory tasks, and tapping (motor) tasks. The following section reviews salient ERP findings reflecting neurocognitive (dys) function in alcoholics and HR offspring of alcoholics and discusses the application of source localization methods (e.g., dipole modeling, current source density [CSD], low-resolution brain electromagnetic tomography [LORETA]) and componential methods (e.g., principal component analysis [PCA], independent component analysis [ICA], trilinear modeling).

## ERP Deficits in Alcoholism

### Sensory and Perceptual Processing (Brainstem Sensory Potentials and P1/P100)

Sensory potentials are the voltage changes recorded in the brain in response to a sensory stimulus, representing the information flow along the pathway from the sense organ to the brain in response to an external stimulus, providing quantitative measures of the functional integrity of the sensory pathways (Zaher 2012). Chronic alcoholics have been reported to have prolonged latency in the auditory brainstem sensory potentials, fast time-locked potentials recorded at the scalp that represent processing along the auditory brainstem pathway (Begleiter et al. 1981; Chan et al. 1985; for a review, see Porjesz and Begleiter 1993). However, these abnormalities in early brainstem components recovered after a period of abstinence (Porjesz and Begleiter 1985) and were not found in HR individuals (Begleiter et al. 1987*a*), suggesting that they are related to the lifetime dose of alcohol consumption (Begleiter et al. 1987*a*; Nicolas et al. 1997). The P1 component of the ERP is a positive-going potential occurring around 100 ms after stimulus onset. P1 represents the basic perceptual processing of the stimulus (Heinze and Mangun 1995) and also is sensitive to various task demands (Taylor 2002). Decreased P1 amplitude (Chan et al. 1986; Maurage et al. 2007; Nicolas et al. 1997), delayed latency (Cadaveira et al. 1991; Chan et al. 1986; Fein et al. 2010), and topographic changes (Miyazato and Ogura 1993) of the P1 component, particularly in visual paradigms, have been observed in chronic alcoholics.

Taken together, early sensory deficits indexed by electrophysiological measures, such as brainstem potentials, seem to be a result of the direct effects of chronic alcohol intake and recover with prolonged abstinence, whereas some later cognitive components, such as P3, do not recover (Porjesz and Begleiter 1985) and may antecede the development of alcoholism (see ERP section on P3).

### Selective Attention (N1/N100)

The N1 or N100 component occurs around 100 ms after the stimulus and represents selective attentional processing; it has been shown to be modulated by the cognitive or emotional salience of the stimulus (Haider et al. 1964; Hansen and Hillyard 1980; Mangun and Hillyard 1995), and a larger N1 component is elicited for the attended and/or salient stimuli (Talsma and Woldorff 2005; Vogel and Luck 2000). Diminished N1 component has been found in both alcoholics (e.g., Cohen et al. 2002; Patterson et al. 1987) and their first-degree relatives (Steinhauer et al. 1987). Whereas the suppressed N1 component in alcoholics and HR study participants may indicate poor attentional modulation during stimulus processing, replication studies with identical methodology are required to confirm the phenomenon of N1-related deficits.

### Automatic Stimulus Change Detection: MMN

Another early occurring ERP component investigated in alcoholism research is the MMN, which is a powerful measure of automatic central auditory processing (Naatanen et al. 2007). MMN is typically evoked by a physically deviant auditory stimulus and occurs between 170 and 240 ms after stimulus onset (Giard et al. 1990), reaching maximal amplitude at frontal scalp locations (Naatanen and Alho 1995). In alcoholism, MMN findings are equivocal. Although some studies reported larger MMN in alcoholics (e.g., Ahveninen et al. 2000) and in HR subjects (e.g., Zhang et al. 2001), others have failed to find any MMN-related changes in alcoholics (Fein et al. 2004*a*,*b*) and in HR individuals (Rodriguez Holguin et al. 1998; van der Stelt et al. 1997). Deficiencies in MMN may be related to deficits in central auditory processing (Naatanen 1995) and impairments in neural systems related to automatic stimulus change detector mechanisms, possibly involving frontal lobes (Alho et al. 1994). More studies are needed to ascertain and characterize the MMN related deficits in alcoholism.

### Error-Related Negativity (ERN/Ne)

Error-related negativity (ERN, or Ne) is a large negative potential observed within 50 to 200 ms (and peaking around 150 ms) after an “incorrect” response in tasks that require “correct” identification of a stimulus presented (Falkenstein et al. 1991; Gehring et al. 1993, 1995; Holroyd et al. 1998). ERN is an electrophysiological index of error monitoring, or detection of the discrepancy between the desired and actually executed action, and is generated in the anterior cingulate cortex (Carter et al. 1998; Debener et al. 2005*b*). Whereas ERN is a preconscious mechanism, a later positive component, termed “error positivity” or Pe, occurring around 300 ms, is related to conscious awareness of the error (Davies et al. 2001; Overbeek et al. 2005). ERN amplitude has been reported to be lower in individuals with schizophrenia (Alain et al. 2002; Bates et al. 2002), opiate dependence (Forman et al. 2004), cocaine dependence/use (Franken et al. 2007; Hester et al. 2007), and externalizing traits such as aggression, bullying, and defiance (Hall et al. 2007), and higher in individuals with obsessive-compulsive disorder (Hajcak and Simons 2002; Johannes et al. 2001) and anxiety traits (Hajcak et al. 2003). Studies have shown that acute alcohol administration significantly reduced ERN amplitude (Bailey et al. 2014; Bartholow et al. 2012; Easdon et al. 2005; Holroyd and Yeung 2003; Ridderinkhof et al. 2002) (for a review on ERN and psychopathology, see Olvet and Hajcak 2008)]. Similarly, heavy drinkers also displayed a smaller ERN amplitude (Bartholow et al. 2012). By contrast, an ERN study (using an error paradigm) in alcoholism reported that ERN amplitudes were increased for alcohol-dependent patients compared with healthy control subjects, particularly in patients with comorbid anxiety disorders (Schellekens et al. 2010). As reviewed in the next sections (as part of N2 and P3 components of ERPs), given the findings that reduced feedback-related negativity (i.e., N2 during loss or gain) in reward paradigms was observed in alcoholics (Kamarajan et al. 2010) and those with a family history of alcoholism (Fein and Chang 2008), more studies are necessary to confirm findings from Schellekens and colleagues (2010) as well as to establish ERN changes in alcohol and other SUDs.

### Attentional Orientation and Conflict Monitoring (N2/N200)

The N2 is a negative going wave observed approximately 200 to 350 ms after stimulus onset, maximally at frontocentral sites, and has been associated with several processes such as the covert orienting of attention, the detection of response conflict (conflict monitoring), response inhibition, and error detection (Jodo and Kayama 1992; Nieuwenhuis et al. 2004; Wijers et al. 1989). Alcoholics have been reported to have longer N2 latency (Cadaveira et al. 1991; Porjesz et al. 1987) and lower amplitude (Cristini et al. 2003; Realmuto et al. 1993) during an oddball task. Decreased N2 amplitude in alcoholics also has been observed during inhibition in a Go/No-Go task (Cristini et al. 2003; Pandey et al. 2012*b*) and during loss in a MGT task (Kamarajan et al. 2010). Further, Fein and Chang (2008) reported that smaller N2 amplitudes in feedback trials were associated with a greater family history density of alcohol problems.

### Target/Context Processing, Inhibitory/Cognitive Control, and Feedback Processing (P3/P300)

The most robust electrophysiological findings in alcoholism are related to the P3 component (Porjesz et al. 2005), a large positive going wave that occurs between 300 and 600 ms after the stimulus (Duncan et al. 2009; Sutton et al. 1965). P3 is not related to the physical characteristics of the stimulus but is related to its “significance” and is an index of various neurocognitive processes, including attention and working memory (Donchin 1981; Kok 2001; Polich 2007; Verleger 1988).

A large body of research has established that alcoholics consistently manifest significantly lower P3 amplitudes under a variety of task conditions and in both genders (for reviews, see Begleiter and Porjesz 1990b; Campanella et al. 2009; Porjesz and Begleiter 1993, 2003; Porjesz et al. 2005). The reduced P3 amplitude in alcoholics does not recover with prolonged abstinence (Porjesz and Begleiter 1985) and has been found to be related to the number of first-degree alcoholic relatives more than the drinking history of an alcoholic (Cohen et al. 1995; Pfefferbaum et al. 1991) or of an HR individual (Benegal et al. 1995). Furthermore, low P3 amplitude in prepubescence has been shown to predict later substance abuse, including alcohol abuse in adolescence (Berman et al. 1993; Hill et al. 1995; Iacono et al. 2002, 2003).

Another body of research shows that, similar to alcoholics, HR offspring manifest significantly lower P3 amplitude under a variety of task conditions (for reviews, see Begleiter and Porjesz 1990*a*,*b*; Porjesz and Begleiter 1990, 1991, 1997; Porjesz et al. 2005). Since the initial report of low P3 amplitudes in young sons of alcoholics prior to any exposure to alcohol (Begleiter et al. 1984), P3 deficits have been reported in both male and female children, adolescent, and young adult HR offspring (cf. Porjesz et al. 2005). It has been hypothesized that reduced P3 reflects underlying neural disinhibition (i.e., hyperexcitability), which in turn may be involved in the predisposition to alcoholism (Begleiter and Porjesz 1999). These findings underscore the utility of P3 as an effective endophenotype in alcoholism. (See more discussion on endophenotypes and genetic findings in a later section, “Electrophysiological Measures as Endophenotypes for Alcoholism.”)

### Language Processing (N4/N400)

N400 is a negative component, occurring around 400 ms (within a 300- to 650-ms window) and predominantly over the centro-parietal scalp region, in response to a semantically incongruent or inappropriate stimulus (for review, see Kutas and Van Petten 1988). N400 in ERP paradigms can be obtained either by presenting sentences with semantically deviant words or by presenting a series of words with a priming effect. A word is responded to more quickly and accurately if it is preceded by similar or related words (primed) than if it follows dissimilar or unrelated words. In normal subjects, unprimed words elicit larger N400s than primed words, whereas N400 for primed words are either small or absent (Kutas and Hillyard 1989). N400 deficits have been reported in several neuropsychiatric and cognitive disorders (Olichney 2013), especially in schizophrenia (Mohammad and DeLisi 2013). In the first study using a semantic priming paradigm in alcoholics, it was reported that alcoholics exhibited an N400 component for both primed and unprimed words, whereas the control subjects elicited N400 only for unprimed words (cf. Porjesz and Begleiter 1995). Using a sentence paradigm, reduced amplitudes in alcohol-dependent subjects (Ceballos et al. 2003, 2005; Nixon et al. 2002) were reported. In a priming study (where some of the words were antonym pairs), Roopesh and colleagues (2010) reported that although control subjects showed significant attenuation of the N400 response to the primed word compared with the unprimed word, alcoholics did not show this differentiation. Similar results of lack of attenuation to primed stimuli were found with the same paradigm in HR offspring (Roopesh et al. 2009). These findings indicate that alcoholics and HR offspring manifest inefficient neural processing, responding similarly regardless of stimulus and task requirements.

## Advances in ERP Methods

### Source Localization Methods

Although brain electrical activity recorded from scalp EEG has high temporal resolution on the scale of milliseconds, the spatial resolution can be limited, as cortical electrical activity is blurred over the scalp when volume conducting through the low conductivity skull (He et al. 2001; Nunez 1981; Srinivasan 1999). Several attempts have been made to improve the spatial resolution of the scalp EEG by using source localization techniques that employ computational algorithms to “de-blur” the recorded scalp potentials. (For a review on methods, see Grech et al. 2008). The most commonly used source localization methods are discussed below.

### Dipole Source Modeling for the ERP Data

Dipole source analysis, as a tool to identify the generation of neuronal structures and to separate overlapping activity, also has been applied to analyze scalp-recorded ERPs. It mainly has been applied to P3(00) data (Hegerl and Frodl-Bauch 1997) to understand the sources of P300 activity (Tarkka et al. 1995) and to separate and enhance the reliability of overlapping sources of P300 subcomponents (Hegerl and Frodl-Bauch 1997). A variety of dipole source analysis methods often are performed using the software brain electrical source analysis (BESA) (Miltner et al. 1994). Dipole modeling techniques permit estimates of underlying brain sources of scalp-recorded potentials, thus helping to interpret ERP findings with respect to those obtained from other methods (e.g., fMRI, PET or brain lesion studies) (cf. Amodio et al. 2014). Dipole source analyses have been implemented to identify sources and deficits of ERP potentials in schizophrenia (Oknina et al. 2005; Youn et al. 2003), depression (Li et al. 2011), anxiety (Li et al. 2011), obsessive-compulsive disorder (Kim et al. 2006, 2009), drug use (Mejias et al. 2005; Tuchtenhagen et al. 2000; Wan et al. 2009), and AUD (Hegerl et al. 1996*a*; Tarkka et al. 2001). In alcoholism, an increase in the intensity dependence (i.e., corresponding amplitude change based on stimulus intensity) of the tangential dipole for the N1/P2 component was observed in alcoholics, whereas a decrease was found in healthy control subjects (Hegerl et al. 1995, 1996*a*). Tarkka and colleagues (2001) performed dipole source analysis of ERPs related to automatic auditory processing (i.e., MMN) and found that processing of alerting tones was located at frontal regions in violent alcoholics, whereas the same processing was identified at medial temporal regions in nonviolent alcoholics and normal subjects. Similarly, dipole modeling has identified changes in the location of brain sources for P50, P100, and MMN components in alcoholics (Pekkonen et al. 1998).

### Current Source Density (CSD)

EEG recorded with scalp electrodes represents summated activity from multiple brain sources and not just the source activity close to the electrode location. An estimate of the local radial current density or CSD for the EEG activity is normally calculated using a surface Laplacian method, an algorithm first implemented by Hjorth (1975), to improve spatial resolution and eliminate the influence of reference electrode distortions. Surface Laplacian reflects the radial projections of underlying current sources within the brain, and represents a unique, unambiguous measure of neuronal activity at scalp by providing estimates of local current flux from the brain through the skull into the scalp (Tenke and Kayser 2012). The surface Laplacian mainly acts as a spatial filter, and provides a more local representation of electrophysiological activity than the directly recorded potential (Hjorth 1975; Nunez 1981; Wang and Begleiter 1999). The CSD creates neuronal generator patterns contributing to scalp-recorded EEG in terms of local sources (positivity that represents the current flow from the brain to the scalp) and sinks (negativity that indicates the current flow from the scalp to the brain) and thus offers insights into the anatomical origins of the scalp potentials (Tenke and Kayser 2012). However, there are many methods for computing surface Laplacians of brain potentials. Local methods interpolate potentials only from the surrounding electrodes (Hjorth 1975), whereas global methods use all the electrodes by constructing a global potential function, so that the Laplacian at any point depends on the potentials at all electrodes. Interpolation can be implemented using the spherical spline method (Perrin et al. 1987). Further information on the methods and algorithms are detailed elsewhere (Nunez 1989; Srinivasan et al. 1996; Wang and Begleiter 1999).

CSD methods, using ERP data, have been successfully applied to several neuropsychiatric conditions, including alcoholism, to elucidate differences in source activations during cognitive processing (Kamarajan et al., 2014, in press). Adult alcoholics manifest low P3-related source activations during the performance of oddball tasks (Cohen et al. 2002; Hada et al. 2000; Rodriguez Holguin et al. 1999*a*) and showed changes in topographic activation patterns related to response inhibition (Kamarajan et al. 2005*a*), reward evaluation (Kamarajan et al. 2012), and language processing (Roopesh et al. 2010). Similar lower activations of P3 sources, as well as differences in CSD topographic patterns, have been reported in HR offspring of alcoholic parents (Hada et al. 2001; Ramachandran et al. 1996; Rodriguez Holguin et al. 1999*b*).

**Figure 1:**
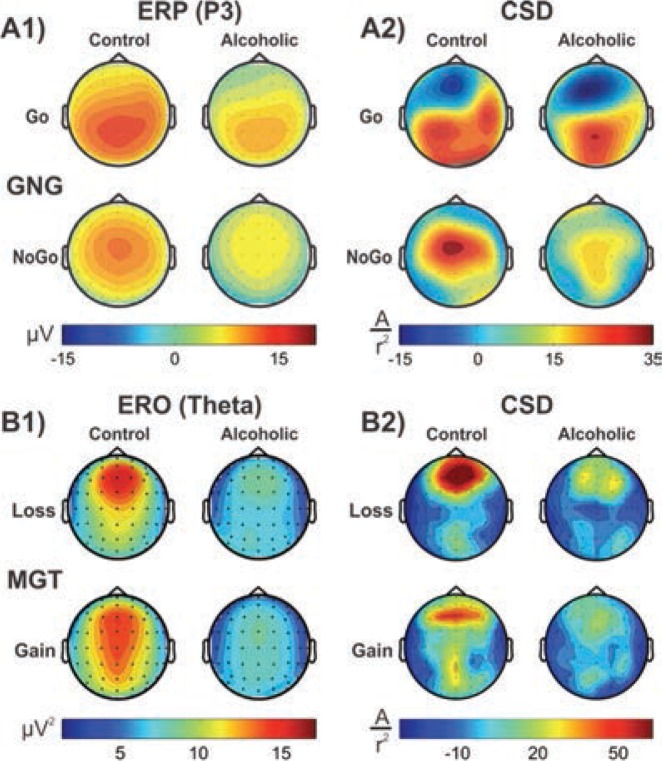
The current source density (CSD) method provides measures of source activations, which are otherwise blurred in the scalp potentials. **A1)** P3 event-related potential (ERP) topography showing lower P3 amplitude (in microvolts) in alcoholics during both Go and No-Go conditions in a Go/No-Go task. **A2)** CSD maps (in ampere per squared radius) showing the Go condition with two bilateral sources in control subjects and only a midline source in alcoholics and illustrating the No-Go condition with a stronger, more focused source over the central region in control subjects and a weaker, more diffuse source over the central and posterior regions in alcoholics (Kamarajan et al. 2005*a*). **B1)** Topography of event-related oscillations (EROs) theta power (in microvolts squared) in alcoholics and control subjects during the loss condition in an monetary gambling tasks (MGT) task, plotted for ERO theta power during the N2-P3 complex (200 to 500 ms). **B2)** CSD maps of ERO theta activity showing a single and stronger midline prefrontal source during the loss condition in control subjects contrasted with bilateral and weaker prefrontal sources in alcoholics; during the gain condition, control subjects had well-defined anterior and posterior sources whereas alcoholics showed weaker and more diffuse sources (Kamarajan et al. 2012).

CSD studies in alcoholism also revealed region-specific activations and altered topographic features. In a visual category-matching task, Ji and colleagues (1999) reported suppressed activations at the left temporal-occipital areas in alcoholics during both matching and nonmatching conditions (around 250 ms). In a Go/No-Go task, Kamarajan and colleagues (2005*a*) found that alcoholics had lower P3 amplitudes and a more diffuse and weaker P3 source without the prefrontal sink, which was observed in the control subjects during the No-Go condition (see figure 1, panels A1 and A2). Further, Kamarajan and colleagues (2012) compared topographic patterns of ERO theta activity representing total theta power with CSD maps computed from theta amplitude data extracted within the time interval of 200 to 500 ms during the feedback of loss and gain during a single-outcome monetary gambling task, with a bet of either 10 cents or 50 cents, and found low theta power and lower CSD activations in alcoholics along with topographic differences between groups (see figure 1, panels B1 and B2).

### LORETA

LORETA is a functional imaging method to localize source activations of the scalp-recorded EEG/ERP potentials by mapping the activations in three-dimensional volume elements (voxels) in the digitized Talairach atlas (Pascual-Marqui et al. 1994). This method has been further elaborated as standardized LORETA or sLORETA (Pascual-Marqui 2002) and exact LORETA or eLORETA, with reportedly improved algorithm and other tools (Pascual-Marqui 2007). The LORETA method has been widely used to understand brain activation patterns during cognitive processing in healthy study participants as well as in several clinical conditions (Pascual-Marqui et al. 2002), as shown in its Web site: http://www.uzh.ch/keyinst/loreta.htm.

Several studies have used LORETA methods to investigate cognitive dysfunction in alcoholics and HR offspring. Prabhu and colleagues (2001) reported that source localization of visual P3 showed decreased activation in female alcoholics compared with control female social drinkers in right dorsolateral prefrontal cortex and ventromedial frontocentral regions. Chen and colleagues (2007) found significantly reduced P3-related current density activation in frontal regions (anterior cingulate, medial, and superior frontal) in alcoholic study participants while processing target stimuli in a visual oddball task. Alcoholics scored higher on impulsivity, and highly impulsive participants had the lowest activations in these areas. In a Go/No-Go task, Kamarajan and colleagues (2005*b*) found that offspring of alcoholics exhibited reduced activation in frontal, anterior cingulate, and temperoparietal regions during the P3 activity of the No-Go condition.

**Figure 2:**
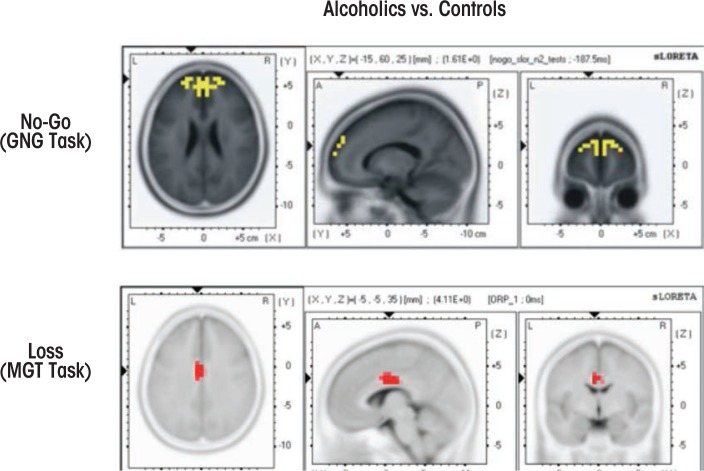
Application of standardized low-resolution brain electromagnetic tomography (sLORETA) to alcoholism. **Top panels:** Current density in alcoholics and control subjects were compared in a Go/No-Go task using sLORETA. Alcoholics showed significantly lower current density activations in bilateral anterior prefrontal regions during No-Go–related N2 activity (yellow blobs in top panels), indicating dysfunctional inhibitory control in alcoholics (Pandey et al. 2012*b*). **Bottom panels:** A sLORETA study in an MGT task found that alcoholics showed decreased current density activation at the middle cingulate cortex region during loss-related P3 activity (red blobs in bottom panels), indicating deficient activation in the reward-related structures or networks (Kamarajan et al. 2010).

Using sLORETA in a Go/No-Go task, Pandey and colleagues (2012*b*) reported significantly smaller N2-related activations during the No-Go condition at bilateral anterior prefrontal regions in alcoholics compared with control subjects (see figure 2). Further, sLORETA analysis in a MGT task revealed that alcoholics, as compared with control subjects, showed significantly lower P3-related current density activations at cingulate gyrus, along with significantly reduced N2-related current density at postcentral gyrus, inferior frontal gyrus, and precentral gyrus during both loss and gain conditions (Kamarajan et al. 2010) (see figure 2). These studies demonstrate the utility of LORETA methods in revealing the activity patterns of key brain regions that are associated with neurocognitive dysfunction in alcoholics and HR offspring.

## Componential Analyses of ERPs

### PCA

The central idea of the principal component analysis is to reduce the dimensionality of a dataset consisting of a large number of interrelated variables, while retaining as much as possible of the variation present in the dataset. This is done by transforming the data into a new set of variables, called the principal components, which are uncorrelated and often orthogonal and which are ordered so that the first few retain most of the variation present in all of the original variables (Jolliffe 2005). The PCA method decomposes the entire ERP dataset into individual elementary curves or components, and the sum of the derived components should approximate the waveform of the measured ERP (Begleiter et al. 1987*b*). PCA components (i.e., factor loadings or factor waveforms), together with their associated weights (i.e., topography of factor scores), can each be represented in terms of their accounted variance and interpreted based on their topographic significance (Kayser and Tenke 2006). Often, the initially derived components are further subjected to factor rotation (e.g., varimax rotation) to achieve/improve factor structure while maintaining factor orthogonality (being perpendicular from each other) (Kayser and Tenke 2003). Studies have shown that PCA has been useful to segregate components or factors from the ERP data and to determine the dimensionality of effects of interest (Chapman and McCrary 1995; Dien and Frishkoff 2005; Pourtois et al. 2008; Van Boxtel 1998). Performing PCA on the Laplacian transformed waveforms as a generic method for identifying ERP generator patterns also offers unique components with sharper, simpler topographies and without losing or distorting any effects of interest (Kayser and Tenke 2006). Further, the PCA approach has been applied to decompose time-frequency components of the ERPs to elicit topographically meaningful oscillatory components (Bernat et al. 2005, 2007*b*).

PCA-based decomposition, along with CSD transformation, has been a useful approach to elicit topographically distinct activation patterns to distinguish clinical groups from control subjects, as applied in schizophrenia (Kayser et al. 2006, 2010) and depression (Tenke et al. 2008, 2010). Using a MGT task, Bernat and colleagues (2011) examined the relationship between externalizing proneness and the feedback-related positivity (FRP/P3) and negativity (FRN/N2). Using PCA decomposed time-frequency measures accompanying P3 response to feedback cues revealed that feedback-locked delta-P3 activity was reduced among individuals high in externalizing proneness, whereas theta-N2 response was unrelated to the externalizing index. Begleiter and colleagues (1987*b*) elicited P3 amplitude differences between HR offspring of alcoholics and low-risk control subjects using PCA-derived ERP waveforms. Using a similar method in a flanker task in an alcohol administration study, Bartholow and colleagues (2003) reported that a PCA-derived frontal negativity ERP component was related to the high dose of alcohol during both correct and incorrect response trials. However, incorrect allocation of components and lack of functionally meaningful components have been cited as weaknesses with these methods (Wood and McCarthy 1984), although some solutions have been suggested to overcome these limitations (Dien et al. 2003).

### ICA

ICA decomposes ERP data into a set of components that are distinct and maximally independent time courses but are not necessarily orthogonal scalp projections (Makeig et al. 1997). In other words, ICA spatially and temporally filters data without the assumption of the orthogonality of components to represent the input data as a sum of temporally independent and spatially fixed components that arise from distinct or overlapping source activations. The ICA method has been demonstrated to extract independent components of early and late ERP potentials that can explain functionally distinct brain processes (Makeig et al. 1999*a*,*b*), and has been applied to a variety of task paradigms involving perceptual, cognitive and emotional processes (Debener et al. 2005*a*; Desjardins and Segalowitz 2013; Iidaka et al. 2006; Matsumoto et al. 2005; Sato et al. 2001; Schevernels et al. 2014).

**Figure 3:**
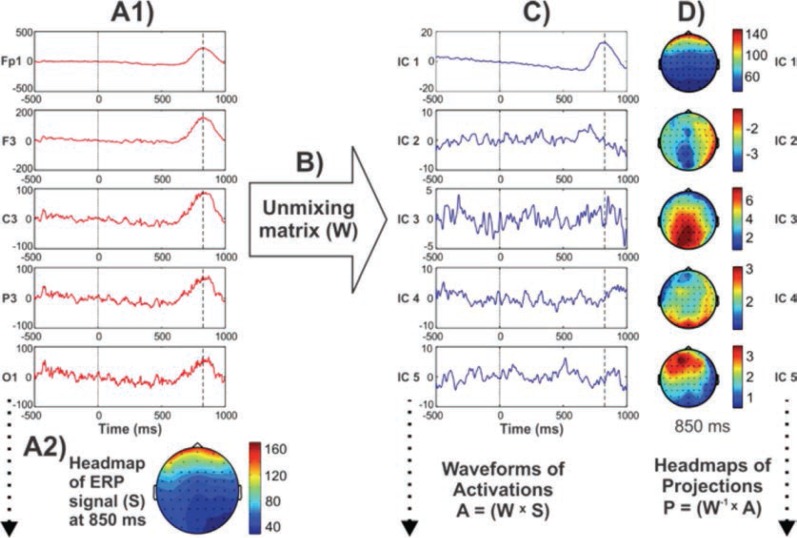
Steps involved in the derivation of independent component analysis (ICA) components in event-related potential (ERP) data, as described by Jung and colleagues (2000, 2001), based on single trials from an ERP dataset from the monetary gambling tasks (MGT) task for illustrative purposes. The waveforms **(panel A1)** and topographic map **(panel A2)** of the ERP signal (S) are shown (in µV) for a trial epoch of an MGT task during the feedback of loss. The “unmixing” matrix (W) **(panel B)** is computed using the ICA algorithm on a “training” dataset (S) representing a larger dataset (e.g., ERP data of adult males during loss condition). “W” consists of weights in a square matrix with the size of number of input channels. The activation matrix **(A)** is obtained by multiplying “W” with “S” **(panel C)**. The rows of “A” represent the time courses of the activations of ICA components. Finally, the “projections” (P) for a given “S” are the product of the inverse matrix of “W” [W-1] and the activations corresponding to the “S” for which ICA components are to be derived **(panel D)**. “P” refers to the relative projection strengths for the respective components at each of the scalp electrodes. It is shown that the EOG activity in the signal (around 850 ms) has been well-captured by the first ICA component. The headmaps have been plotted for 850 ms post-stimulus where the EOG occurs. The 0 (zero) ms on the X-axis of the waveform plots represent the onset of a feedback signal. Downward arrows represent the continuation of the process for remaining electrodes or components.

Processing steps involved in the derivation of ICA components are illustrated in figure 3, following the method described by Jung and colleagues (2000), visually demonstrating ICA’s ability to capture the massive electroocculogram^5^ (EOG) activity in the resulting component(s), although its use in decomposing meaningful components underlying ERP components have been illustrated elsewhere (Makeig and Onton 2009; Makeig et al. 1999*a*,*b*, 2004). These spatially “independent” components are thought to be suggestive of their physiological origins (e.g., eye activity projects mainly from frontal sites and progresses toward posterior sites) (Jung et al. 2001). When these resultant components are combined or “remixed,” the original “composite” signal can be obtained.

Whereas some functionally meaningful components help explain the contribution of specific topographic activity patterns in the ERP time course (Makeig et al. 1999*a*,*b*), one or more of the ICA components that are not related to brain processes (i.e., ocular, cardiac, and muscular artifacts) can be removed (Iriarte et al. 2003). ICA algorithms have been used to identify topographic patterns of ERPs associated with specific diagnostic categories, such as mild cognitive impairment (Li et al. 2013; Missonnier et al. 2013*b*) and voluntary hypoxic state (Menicucci et al. 2013). In alcoholism research, Olbrich and colleagues (2002) studied ICA-derived spatial components of ERPs in a visual CNV paradigm and found increases in the ICA components of N2 and negative slow waves as well as decreases in P3 in alcoholics compared with control subjects. Evidence suggests that ICA is becoming a useful signal processing method for analyzing electrophysiological data, and may become an important tool in alcoholism research as well.

### Trilinear Modeling

Componential methods such as PCA and ICA estimate the individual spatial and temporal components for a given subject and a given condition separately and do not allow the simultaneous comparison of ERP components across subjects and conditions (Wang et al. 2000). Researchers therefore developed trilinear modeling, a novel method for estimating a set of spatial components (brain maps) and temporal components (waveforms) of time-locked brain potentials across subject groups and task conditions (Wang et al. 2000). Trilinear modeling is one member of a family of modeling techniques that extends two-dimensional linear modeling to multidimensional modeling, in general known as N-way modeling. Trilinear modeling is based on the topographic component model (TCM) (Mocks 1988), which models brain potentials in a trilinear form. The trilinear approach builds on singular value decomposition (SVD) and extends the TCM mainly by replacing the diagonal amplitude matrix by a general loading matrix and by allowing the number of spatial and temporal components to be different (Wang et al. 2000). Thus, the trilinear model has the advantages of both SVD and TCM methods. The trilinear components are uniquely determined and more interpretable. Trilinear modeling can be used for interindividual comparison studies, single-trial modeling, clinical classification of patients, and data filtering. For example, the trilinear method was applied in “dynamic time warping” to align the repeated single trials of the ERPs in order to eliminate the timing differences and to get an improved estimate of the ERP components (Wang et al. 2001). In their original work, Wang and colleagues (2000) had demonstrated the decomposition of visual P3 into 16 spatio-temporal components. Significant linkage between time-warped P3-related trilinear components in a visual oddball paradigm in densely affected alcoholic families from the Collaborative Study on the Genetics of Alcoholism (COGA) has been reported (Porjesz et al. 2002*b*).

The trilinear decomposition method also has been used for resting EEG, to estimate spectral and spatial components. These trilinear components of the resting EEG have been used in a COGA study to reduce multiple testing of electrodes and frequency bands, where significant linkage/linkage disequilibrium and association was found between a trilinear beta EEG phenotype and *GABRA2*, a GABA_A_ receptor gene, later found to be also associated with alcoholism (Edenberg et al. 2004; Porjesz et al. 2002*a*). (See the section “Electrophysiological Measures as Endophenotypes for Alcoholism.”) Trilinear decomposition also has been applied in several studies with EEG (Martinez-Montes et al. 2004; Miwakeichi et al. 2004) and EROs (Morup et al. 2006, 2008). Recently, Verleger and colleagues (2013) applied trilinear decomposition to understand the relationship between CNV and the P3 complex in a Go/No-Go paradigm and obtained relevant components. Trilinear decomposition also has been successfully applied to seizure localization and found to be more sensitive than visual interpretation of the EEGs recorded during a seizure (De Vos et al. 2007). Trilinear modeling has great utility in alcoholism, and further studies are currently being conducted.

## EROs: Findings and Prospects

EROs are time-frequency measures of brain electrical activity that are temporally associated with a sensory or cognitive event (Basar et al. 1999, 2001). According to Basar and colleagues (1999), selectively distributed delta, theta, alpha, and gamma oscillatory systems mediate resonant communication networks through large populations of neurons during cognitive processing. The “phase reset model” suggests that ERPs are generated by the resetting of ongoing brain oscillations in response to a neurocognitive event (for a critical discussion, see Sauseng and colleagues 2007). EROs can be classified as (1) “evoked” or phase-locked oscillations, (2) “induced” or non–phase-locked oscillations, and (3) “total” or the summated activity of evoked and induced oscillations (Jones et al. 2006*b*; Tallon-Baudry and Bertrand 1999).

### ERO Findings in Alcoholism

EROs provide a useful method to investigate brain dysfunction in alcoholism and risk. Furthermore, they provide powerful quantitative endophenotypes that have been successfully used to identify genes involved in alcoholism (see the section “Electrophysiological Measures as Endophenotypes for Alcoholism”). Several studies have explored EROs in alcoholics as well as HR offspring or relatives of alcoholics, and the key findings are reviewed below.

### Delta and Theta EROs

**Figure 4:**
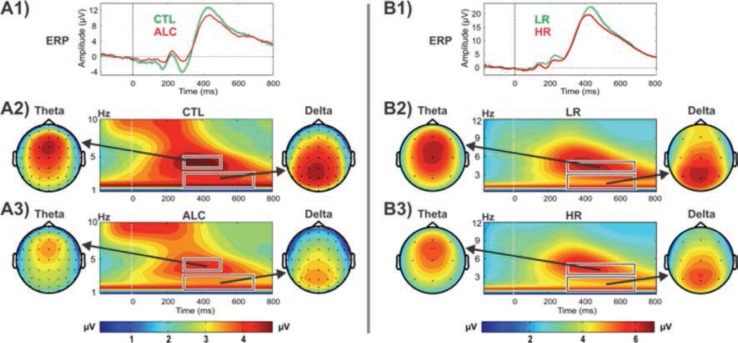
Application of event-related potentials (ERPs) and event-related oscillations (EROs) in alcoholism during a visual oddball task (Jones et al. 2006*b*; Rangaswamy et al. 2007). The left side of the figure **(panels A1–A3)** compares alcoholics (ALC) and control subjects (CTL) (Jones et al. 2006*b*), whereas the right side of the figure **(panels B1–B3)** compares high-risk (HR) offspring and low-risk (LR) control subjects (Rangaswamy et al. 2007). Alcoholics showed lower P3 amplitudes than control subjects **(panel A1)**, whereas HR offspring showed lower P3 amplitudes to targets than LR in the same visual oddball paradigm **(panel B1)**. Panel A2 illustrates time-frequency (TF) plots for control subjects (center rectangular panel) with accompanying topographical head plots for delta (1 to 3 Hz) at the Pz electrode (right) and theta (4 to 5 Hz) at the Fz electrode (left). **Panel A3** illustrates corresponding TF plots for alcoholics (center rectangular panel) with accompanying topographical head plots for delta (right) and theta (left). Alcoholics showed lower activation of both delta and theta EROs compared with control subjects **(panels A2–A3)** during the processing of targets. **Panel B2** illustrates TF plots for LR (center rectangular panel) with accompanying topographical head plots for delta (1 to 3 Hz) at the Pz electrode (right) and theta (4 to 5 Hz) at the Fz electrode (left). **Panel B3** illustrates corresponding TF plots for HR (center rectangular panel) with accompanying topographical head plots for delta (right) and theta (left). Similar to the alcoholics, HR offspring manifested lower activation in P3 **(panel B1)**, delta and theta EROs (**panels B2–B3)** compared with LR control subjects.

**Figure 5:**
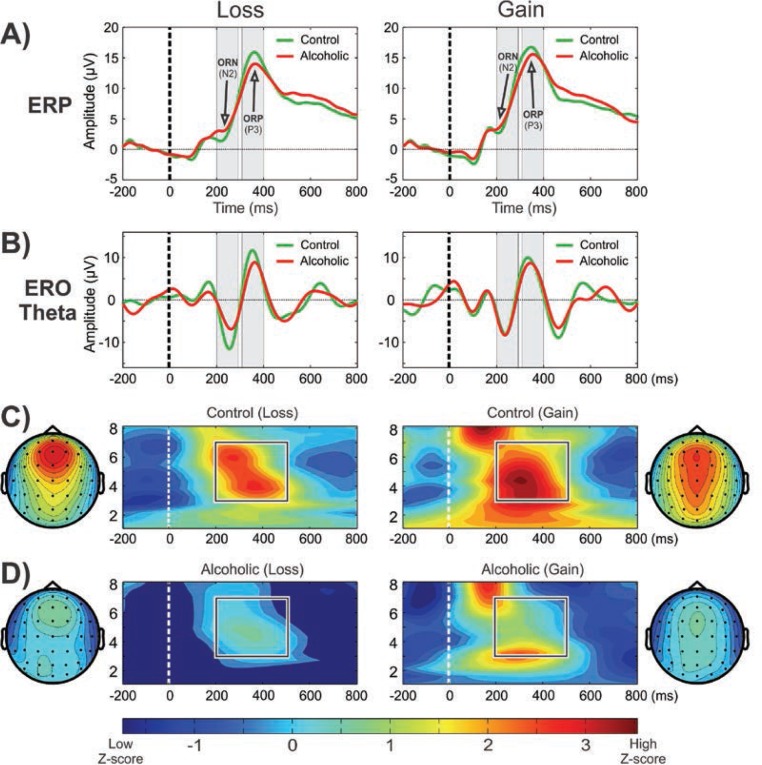
Application of event-related potentials (ERPs) and event-related oscillations (EROs) to alcoholism in a monetary gambling task (MGT) (Kamarajan et al. 2012). **A)** Alcoholics showed lower P3 amplitude of the ERP during loss and gain conditions than control subjects. **B)** ERO theta activity (3 to 7 Hz) was lower during the N2 and P3 time window in alcoholics compared with control subjects. **C)** Time-frequency plots (center panel) and topographic head plots of theta power in control subjects during loss (left) and gain (right) conditions. **D)** Time-frequency plots (center panel) and topographic head plots of theta power in alcoholics during loss (left) and gain (right) conditions. Theta power was lower in alcoholics during loss and gain conditions compared with control subjects

Studies have demonstrated that P3 responses are not unitary phenomena but primarily are the outcome of theta and delta oscillations elicited during stimulus processing (Basar-Eroglu et al. 1992; Karakas et al. 2000*a*,*b*); theta oscillations have a more anterior topography and are maximal over frontal areas, whereas delta oscillations have a posterior topography and are maximal over parietal areas. ERO delta responses are assumed to mediate signal detection, decision-making, and context/reward processing (Basar 1999*b*; Basar et al. 2001; Kamarajan et al. 2004; Schurmann et al. 2001), whereas ERO theta rhythms are associated with conscious awareness, episodic retrieval, recognition memory, executive control, inhibitory processing, and reward processing (Basar et al. 2001; Doppelmayr et al. 1998; Kamarajan et al. 2004, 2008; Karakas et al. 2000*b*; Klimesch et al. 2001). Studies consistently have found that alcoholics and their HR off-spring showed decreased delta and theta ERP power during oddball, Go/No-Go, and MGT tasks (for reviews, see Pandey et al. 2012*a*; Porjesz et al. 2005; Rangaswamy and Porjesz 2008*b*) (see figures 4 and 5).

### Gamma Band EROs

Gamma oscillations during cognitive tasks are thought to be involved in selective attention and feature binding (Bertrand and Tallon-Baudry 2000; Fell et al. 2003; Tallon-Baudry et al. 1996). According to Fries and colleagues (2007), gamma rhythm may serve as a fundamental computational mechanism for the implementation of a temporal coding scheme that enables fast processing and flexible routing of activity during signal processing, by supporting fast selection and binding of distributed responses. Particularly, early phase-locked gamma is involved in the selection/identification of target stimuli and represents top-down mechanisms during selective attention (Fell et al. 2003). Neuroimaging studies have identified fronto-parietal attentional networks that may subserve the top-down control of selective attention (Corbetta et al. 2000; Giesbrecht et al. 2003). This early evoked gamma activity has been reported to be larger to attended (target) compared with unattended (non-target) stimuli, suggesting a top-down control mechanism (Busch et al. 2006; Debener et al. 2003; Yordanova et al. 2002).

Studies have found that early evoked gamma activity was abnormal (either higher or lower) in patients with psychiatric disorders (e.g., Basar-Eroglu et al. 2007; Ozerdem et al. 2010; Yordanova et al. 2001). In abstinent alcoholics, researchers have reported a significantly reduced gamma band (28 to 45 Hz) response (0 to 150 ms) in the frontal region during target stimulus processing in a visual oddball task (Padmanabhapillai et al. 2006*a*). Similar reductions in early gamma response also have been found in children of alcoholics (ages 7–17 years) at the posterior regions (Padmanabhapillai et al. 2006*b*). The regional variation in gamma differences observed in children of alcoholics compared with adult alcoholics could be attributed to the fact that the frontal lobes still are in the process of maturation in children and adolescents (Sowell et al. 2004). These deficits further emphasize the view that alcoholism may be associated with deficient frontal (top-down) processing and a dysfunctional fronto-parietal attentional network (Goldstein and Volkow 2011; Rangaswamy et al. 2004*a*).

## Advances in ERO Methods

### Event-Related Desynchronization and Synchronization (ERD/ERS)

ERD/ERS is a valuable technique to unravel time–frequency–space dynamics of cortical oscillations across brain regions during cognitive and motor processing (Klimesch et al. 1997; Krause 2006; Pfurtscheller 1999, 2001; Pfurtscheller and Aranibar 1979). According to Pfurtscheller (2001), ERD represents an activated cortical area with increased excitability, whereas ERS indicates a deactivated cortical area with decreased excitability. Specifically, ERD represents the percentage of decrease, whereas ERS indicates an increase in band power during an event as compared with power in a baseline window (Doppelmayr et al. 1998).

The ERD/ERS method has been useful in understanding cognitive processing abnormalities in several clinical conditions, such as schizophrenia (Bachman et al. 2008; Fujimoto et al. 2012; Xu et al. 2013), attention-deficit hyperactivity disorder (Missonnier et al. 2013*a*), Alzheimer’s disease (Babiloni et al. 2000; Karrasch et al. 2006), Parkinson’s disease (Dushanova et al. 2010; Ellfolk et al. 2006; Labyt et al. 2003), and epilepsy (Houdayer et al. 2012; Pfurtscheller et al. 2003; Visani et al. 2011). A few studies have investigated the acute effects of alcohol on brain oscillatory responses. Krause and colleagues (2002) studied alcohol-induced alterations in ERD/ERS during an auditory memory task and found that alcohol decreases alpha-ERS responses during encoding and increases alpha-ERD responses during recognition. In an alcohol-approach avoidance task, Korucuoglu and colleagues (2014) found that acute alcohol facilitates response preparatory processes for approach alcohol trials in social drinkers. Posterior beta-ERD was found to increase during preparation for alcohol-approach trials, whereas the beta-ERD in the congruent block increased following alcohol administration. Studies using ERD/ERS measures in alcoholism are currently being conducted.

## Connectivity Measures During Task-Related Conditions

### ERO Coherence

Coherence is an estimate of the consistency of relative amplitude and phase between two signals within a frequency band and represents functional interactions across brain regions (see the earlier section “Resting EEG Coherence”). When this coherence function is measured with the same algorithm but using signal processing techniques to extract time-frequency measures (e.g., EROs with S-transform, matching pursuit, wavelet transform, etc.) during a cognitive task, it represents functional connections between neural systems associated with specific cognitive activity (Qassim et al. 2013; Sakkalis 2011). This linear coherence measure generally is distinct from phase synchronization or phase synchrony (Lachaux et al. 1999), which refers to the method that measures phase locking (i.e., level of phase alignment) between signals oscillating at the same frequency (see the next section for details). Thus, ERO coherence is a linear function computed instantaneously by applying time-frequency analysis, such as wavelet analysis, to activity during a task (Torrence and Compo 1998). Using the coherence method, studies have identified possible dysfunction in connectivity between brain regions in several neuropsychiatric conditions (for reviews, see Basar 2013; Sakkalis 2011; Yener and Basar 2013). Diminished event-related gamma band coherence has been reported in schizophrenia (Sakkalis et al. 2006) and bipolar disorder (Ozerdem et al. 2011). In alcoholics, a recent study (Ismaili et al. 2012) found significantly increased wavelet coherence in theta (4 to 8 Hz), alpha (8 to 13 Hz), and gamma (50 to 60 Hz) bands at frontal and occipital regions during 100 to 200 ms poststimulus while performing a visual discrimination task. More alcoholism studies applying this method are under way.

### ERO Phase Synchronization

Phase synchronization is a measure of phase locking between two signals (Lachaux et al. 1999) and represents a mechanism for long-range neural integration involving interactions between the participating local networks (Varela et al. 2001). In event-related data, phase synchronization quantifies the phase differences between the signals across trials (phase-locking factor) by extracting the instantaneous phase of each signal at the specified (target) frequency (Lachaux et al. 1999). Phase-locking factor (also called intertrial phase coherence) is a measure of phase consistency across trials from a single electrode or source (Delorme and Makeig 2004). The phase synchronization method assumes that two dynamic systems may have their phases synchronized, even if their amplitudes are zero correlated (Mormann et al. 2000; Sakkalis 2011). Thus, phase synchronization measures the similarity of two time series (signals) in terms of phase consistency or phase-locking factor and varies in value between 0 (no synchronization) to 1 (perfect synchronization) (Lachaux et al. 1999; Tallon-Baudry et al. 1996). During the processing of cognitive tasks, the phase-locking index varies based on task conditions, brain regions, and frequency bands. For example, Kolev and colleagues (2001) investigated phase locking during passive listening to repeated stimuli and active counting of target stimuli and found condition-specific phase-locking indices of alpha oscillations. Similarly, using a Go/No-Go task, Muller and Anokhin (2012) reported that the phase-locking index and phase synchronization were the highest in the Go and No-Go conditions, intermediate in the warning condition, and the lowest in the neutral condition of the task and elicited distinct, dynamic functional networks for response inhibition and execution.

Although the linear coherence measure does not separate the effects of amplitude and phase in the interrelations between the signals, phase synchronization also yields the phase information, which is important to understand the event-related brain dynamics (Lachaux et al. 1999). Dysfunction in phase synchronization during information processing has been reported in several clinical conditions (for a review, see Uhlhaas and Singer 2006), such as schizophrenia (Csukly et al. 2014; Griesmayr et al. 2014; Perez et al. 2013), depression (Olbrich et al. 2014), obsessive compulsive-disorder (Olbrich et al. 2013), and externalizing disorders (antisocial behavior, attention deficit hyperactivity disorder, and substance dependence) (Burwell et al. 2014). In alcoholism, Sakkalis and colleagues (2007) reported that alcoholics showed impaired synchronization and loss of lateralization, most prominently in alpha- and lower beta–frequency bands, during mental rehearsal of pictures. Studies are under way to elucidate further oscillatory dynamics underlying cognitive (dys)function in alcoholics and in HR subjects.

## Granger Causality Analysis

When applied to brain signals, Granger causality as a statistical method measures the degree of predictability of temporal changes in one brain region that can be attributed to those in another region (Bressler and Menon 2010). According to Granger (1969), causal influence can be explained in terms of stochastic (random) processes when the predictability of one process at a given time point is improved by including measurements from the other. Whereas the coherence methods yield only the strength (but not the direction) of the connection, Granger causality can show both strength of connection and directionality for stationary signals. Thus, this method is suitable for the study of directional influences and pathways in neural networks using both frequency and time domains of ERO data (cf. Brovelli et al. 2004).

Granger causality has been successfully used to identify coupling (connectivity) and information exchange across brain regions in a variety of clinical conditions, such as developmental dyslexia (Ligges et al. 2010), epilepsy (Adhikari et al. 2013; Chavez et al. 2003), and Alzheimer’s disease (Dauwels et al. 2009, 2010). Studies are being conducted using Granger causality to understand the directionality of the neural pathways across brain regions involved in neural processing in alcoholics and their HR offspring.

## Potential for Translational Applications of Electrophysiological Measures of Brain Function

Electrophysiological measures and techniques have clinical applications in several important areas, including genetics/endophenotypes, and to inform the fields of diagnostic classification, prevention, response to treatment, cognitive remediation, neurofeedback, and deep brain stimulation (DBS). Current clinical applications and future translational potential of electrophysiological assessments, especially in the context of alcoholism, are discussed below.

### Electrophysiological Measures As Endophenotypes for Alcoholism

Risk for alcoholism is complex and influenced by both genetic and environmental influences and their interactions: multiple genes, each with small effect, phenotypic complexity and heterogeneity, environmental variability, gene–gene interactions, and gene-by-environment interactions (Porjesz and Rangaswamy 2007). It is difficult to find genes affecting complex diseases such as alcoholism and to use diagnosis as the sole phenotype (Tsuang and Faraone 2000). One effective strategy to find genes is the “endophenotype” approach, first proposed by Gottesman and Shields (1972), who defined an endophenotype as an intermediary measure of neuropsychiatric functioning correlated with the main trait of interest and involved in the pathway between genotype and outcome of interest (Gottesman and Gould 2003). An effective endophenotype must meet three important criteria: (1) it is associated with the illness in the population (i.e., present in affected individuals); (2) it is heritable; and (3) it is found in unaffected relatives of probands at a higher rate than in the general population (including offspring before the onset of the illness). Neurophysiological quantitative measures that meet these three criteria can serve as effective endophenotypes. That is, they can help identify genes associated with the disorder and elucidate mechanisms that may improve understanding of the disorder. Specifically, only heritable electrophysiological measures that differentiate alcoholics from nonalcoholics are used as endophenotypes, to be sure that the measure is related to the disorder (alcoholism). Furthermore, the neurophysiological measure must be able to differentiate between HR offspring of alcoholics and low risk offspring of non-alcoholics (controls) who have no first or second degree alcoholic relatives, and are not at high risk to develop alcoholism (Porjesz and Rangaswamy 2007). These highly heritable and quantitative measures are closer to the gene function, and several measures (e.g., beta power and theta coherence of resting EEG, P3 amplitude and related theta and delta EROs during the oddball task) have been successfully used to identify genes associated with risk for alcoholism and related disorders (for reviews, see Porjesz and Rangaswamy 2007; Rangaswamy and Porjesz 2008*a*,*b*).

One EEG measure, the beta rhythm (i.e., beta 1 [12.5 to 16 Hz], beta 2 [16.5 to 20 Hz], and beta 3 [20.5 to 28 Hz] bands) of the resting EEG, meets criteria as an endophenotype. Beta power is highly heritable (86 percent) (van Beijsterveldt et al. 1996) and is increased in alcoholics (e.g., Bauer 2001; Rangaswamy et al. 2002) and HR offspring (e.g., Pollock et al. 1995; Rangaswamy et al. 2004*b*). Enoch and colleagues (2003) found that LVA in female subjects was associated with a genetic variant that leads to low activity in COMT, the enzyme that metabolizes dopamine and norepinephrine (NE), leading the researchers to hypothesize that altered NE levels may be related to LVA, anxiety, and alcoholism. Beta power has been found to have a genetic link and association with *GABRA2*, a receptor gene for GABA_A_ (Edenberg et al. 2004; Porjesz et al. 2002*a*). Beta rhythm is attributed to a balance between networks of excitatory pyramidal cells and inhibitory interneurons involving GABA_A_ action as the pacemaker (Whittington et al. 2000). The increased beta in alcoholics and HR offspring indicates an imbalance in excitation-inhibition (CNS disinhibition) that precedes the development of alcoholism and may be an index of a predisposition to it (Porjesz et al. 2005). Association of the *GABRA2* receptor gene with a diagnosis of alcohol dependence originally was reported in the COGA study (Edenberg et al. 2004) and replicated by many other studies worldwide (Covault et al. 2004; Fehr et al. 2006; Lappalainen et al. 2005; Philibert et al. 2009; Soyka et al. 2008). In COGA, it has been found that the association with *GABRA2* in adults was strongest in alcoholics who were more severely affected and in those who also had comorbid SUDs (Agrawal et al. 2006). In children, *GABRA2* was found to be associated with conduct disorder, a precursor phenotype (Dick et al. 2006). The heritability of EEG coherence has been examined in twin and family studies (Chorlian et al. 2007; van Baal et al. 2001; van Beijsterveldt et al. 1998). Further, in COGA, a high theta-coherence phenotype has been found to be linked and associated with two inhibitory neurotransmitter receptor genes: *GABRA2*, and *CHRM2*, a muscarinic acetylcholine receptor gene (Porjesz and Rangaswamy 2007; Rangaswamy and Porjesz 2008*a*). Taken together, this endophenotype approach presents a biological hypothesis relating underlying CNS disinhibition to a genetic risk for alcoholism and related disorders. Variations in GABA_A_ receptor genes influence neural excitability and an imbalance in excitation-inhibition, manifesting as increased beta activity (hyperexcitability or CNS disinhibition) in alcoholics and HR offspring, which in turn may be involved in the predisposition to develop AUD and related disinhibitory disorders. This supports the hypothesis originally proposed by Begleiter and Porjesz (1999).

In addition to resting-state EEG endophenotypes, P3-related measures during cognitive tasks (e.g., oddball task) have been successfully used as endophenotypes to identify genes related to alcoholism. Chen and colleagues (2010) reported significant associations between the P3 amplitude to visual targets as well as to alcohol dependence diagnosis with multiple single nucleotide polymorphisms (SNPs) in the corticotrophin-releasing hormone receptor 1 (*CRHR1*) gene, which has been shown to have a role in the environmental stress response in ethanol self-administration animal models.

One neurophysiological measure that has been successfully used as an endophenotype in identifying several genes associated with alcoholism is the frontal theta ERO during P3 to targets during a visual oddball task in COGA (Chen et al. 2009; Jones et al. 2004, 2006*a*; Kang et al. 2012; Zlojutro et al. 2011). Genetic linkage and association with a muscarinic acetylcholine receptor M2 (*CHRM2*) and frontal theta and posterior delta EROs underlying P3 were reported (Jones et al. 2004, 2006*a*). SNPs in *CHRM2* have been found with comorbid alcohol dependence and depression (Wang et al. 2004) and comorbid alcohol and drug dependence (Dick et al. 2007). Significant linkage and association were reported for the *CHRM2* gene and a spectrum of externalizing disorders in the COGA study (Dick et al. 2008). Luo and colleagues (2005) replicated these findings of an association with *CHRM2*, for alcohol dependence, drug dependence, and affective disorder. Significant linkage and association with *CHRM2* also was found with high theta (6 to 7 Hz) interhemispheric coherence. This high theta interhemispheric coherence also was linked and associated with *GABRA2*. Both GABAergic and cholinergic systems are important in local inhibitory circuits essential for cortical synchronization in the theta band (Porjesz and Rangaswamy 2007). M2 receptors are concentrated in the forebrain and have an inhibitory role in the generation of theta and delta EROs via inhibition of presynaptic release of acetylcholine (Frodl-Bauch et al. 1999). P3 production requires both inhibition of irrelevant networks and activation of relevant ones, and it is likely that *CHRM2* affects the inhibition of irrelevant networks during P3 tasks. In Alzheimer’s disease, where there is degeneration of cholinergic neurons in the nucleus basalis, abnormal theta delta and P3 have been reported. Results from the COGA study showed that delta EROs and *CHRM2* affect the onset of regular alcohol use and alcohol dependence during adolescence and young adulthood (Chorlian et al. 2013). Hill and colleagues (2013) used group-based trajectory modeling of auditory P3 data collected longitudinally from offspring in families with and without familial risk for AUD and found that specific trajectories of P3 were associated with familial risk and *CHRM2* variation, with high familial risk in male offspring. These findings underscore the utility of P3-related measures as effective endophenotypes in genetic studies of psychiatric disorders.

Under the same linkage peak as the *CHRM2* gene, a metabotropic glutamate receptor gene (GRM8) was found to be associated with theta EROs to target stimuli at frontal, central, and parietal regions. The same SNPs were found to be significantly associated with 1CD-10 (World Health Organization 1992) based alcohol dependence (Chen et al. 2009). The neurochemical basis of the target stimulus response—P3 and related theta and delta rhythms—is triggered by glutamatergic activity and modulated by both cholinergic and GABAergic sources (Frodl-Bauch et al. 1999). These same GABAergic, cholinergic, and glutamatergic receptor genes also were found to be associated with alcoholism-related phenotypes. Thus, the same genes initially identified as associated with electrophysiological endophenotypes also were found to be associated with alcoholism-related phenotypes.

In a family-based genome-wide association study, Kang and colleagues (2012) used the same neurophysiological phenotype (frontal theta ERO in the visual oddball task) and found genome-wide significant association with several SNPs in KCNJ6, a gene that encodes the protein G-protein inward-rectifying potassium channel 2 (GIRK2). GIRK2 is widely distributed in the brain and is important in dopaminergic, cholinergic, GABAergic, and glutamatergic synapses (Saenz del Burgo et al. 2008). GIRK2-receptor activation contributes to slow inhibitory postsynaptic potentials that modulate neuronal excitability and therefore is important in regulating excitability of neuronal networks. GIRK2 also is important in alcoholism studies, as it is directly activated by alcohol (Aryal et al. 2009; Blednov et al. 2001; Bodhinathan and Slesinger 2013; Hill et al. 2003; Kobayashi et al. 1999; Lewohl et al. 1999). In addition, GIRK2 receptors are important effectors in both opioid- and alcohol-induced pain relief (Ikeda et al. 2002) and are viable drug targets (Kobayashi et al. 2004; Lotsch and Geisslinger 2011).

These findings further underscore the utility of electrophysiological and neurogenetics in understanding the genetics of alcoholism. Recent and future advances in genetic technology hold promise to enhance our understanding of the pathophysiology of AUD as well as to identify potential targets (e.g., neurotransmitter systems and pathways) for drug discovery for prevention and treatment of alcoholism and related disorders (Bodhinathan and Slesinger 2014).

## Clinical or Translational Aspects of Electrophysiological Measures of Brain Function

### Diagnostic Classification and Subtyping

Quantitative electrophysiological measures have been used to classify patients into diagnostic categories and to identify subtypes within a diagnostic category (Bernat et al. 2007*a*; John et al. 2007; Karaaslan et al. 2003; Prichep et al. 2002). In alcoholism, Branchey and colleagues (1988) found that decrements in P3 amplitude characterized a subgroup of alcoholics with disordered regulation of aggression. Bauer (1994) reported that resting EEG absolute beta power (13.2 to 27.6 Hz) at the vertex (Cz electrode) was observed more in relapse-prone patients than in abstinence-prone patients and control subjects. Bauer (1997) also found that P3 could discriminate multiple subgroups within alcoholism: (1) there was more reduction in visual P3 amplitudes at frontal electrode sites among patients with antisocial personality disorder (ASPD), relative to ASPD-negative patient and control groups; (2) the frontal P3 decrement was significantly correlated with the number of childhood conduct disorder symptoms but not with the presence/absence of a family history of alcoholism; and (3) discriminant function analysis revealed that P3 amplitude alone accurately identified 70.6 percent of the patients who later relapsed and 53.3 percent of the patients who did not. Hegerl and colleagues (1995) recorded N1 and P2 components to auditory stimuli in five different intensities in hospitalized alcoholic patients after 1 week of withdrawal and found that patients with antisocial tendencies showed a significantly stronger intensity dependence of their evoked responses of primary auditory cortices (tangential dipoles). This suggests that alcoholics with strong intensity dependence in their ERPs, along with antisocial tendencies, formed a subgroup with a serotonergic hypofunction and may respond favorably to relapse prevention with serotonergic drugs. Furthermore, Winterer and colleagues (2003) found that increases in bilateral, intrahemispheric posterior coherences in the alpha and beta frequency in alcohol-dependent study participants covaried with depressiveness. Taken together, these findings suggest that electrophysiological measures of brain function can aid in diagnostic classification and subtyping, which may lead to better prevention and treatment strategies.

## Prevention, Response to Treatment or Medications

Neurophysiological measures can potentially aid in prevention strategies. In a comprehensive review on trait markers for alcoholism, Farren and Tipton (1999) offer the possibility that electrophysiological markers, such as low EEG response to alcohol (e.g., Volavka et al. 1996) and reduced P3 wave (Porjesz et al. 2005), are good predictors of the development of later substance abuse in predisposed youths. The authors suggest that these measures therefore are potentially viable tools for identifying subgroups of vulnerable individuals and might be implemented in alcoholism prevention programs. Some of these electrophysiological measures have offered established tools to compare clinical outcomes, such as response to medication or treatment, in several psychiatric disorders (Hegerl and Herrmann 1990; Prichep and John 1992; Suffin and Emory 1995), including schizophrenia (Knott et al. 2000), depression (Bruder et al. 2008, 2013; Cook et al. 2005), attention-deficit hyperactivity disorder (Arns et al. 2008; Chabot et al. 1999), and alcoholism (Cristini et al. 2003; Saletu-Zyhlarz et al. 2004). For example, Ford and colleagues (1986) measured EEG coherence in individuals with paranoid schizophrenia, dysthymia, and affective disorder who received tricyclics, neuroleptics, or no medication and found that coherence values were highest in paranoid schizophrenics, decreased with neuroleptic medication, and increased with tricyclic antidepressants. Similarly, Hegerl and colleagues (1992) found that responders to prophylactic lithium medication in affective psychosis showed a steeper slope of the amplitude/stimulus-intensity function (ASF slope) in N1 and P2 components than in nonresponders. The pronounced amplitude increases in the Loudness Dependence of the Auditory Evoked Potential (LDAEP), such as tangential dipole activity and N1-P2 components with increasing stimulus intensity (loudness), have been proposed as an indicator of a low serotonergic neurotransmission. This feature of augmented LDAEP has been observed during the alcohol-intoxicated state (Hegerl et al. 1996*a*) and after the intake of acamprosate during treatment (Hegerl et al. 1996*b*). Further, Pillay and colleagues (1996) showed that female patients with abnormal EEGs before starting the clozapine treatment had a significantly greater improvement in global assessment of functioning scores compared with female patients with normal EEGs. These results suggest that electrophysiological measures are useful to predict a clinical response in specific groups of patients.

In alcoholism, Cristini and colleagues (2003) found that the P300 amplitudes to targets in an auditory oddball paradigm as well as in a CNV paradigm were significantly higher among patients who relapsed during the 3-month follow-up than in those who remained abstinent. Saletu-Zyhlarz and colleagues (2004) compared EEG profiles of relapsing patients with those of abstaining patients during 6 months of pharmacologically supported relapse prevention therapy. Aberrant brain function, characterized by a decrease in delta and slow alpha power and an increase in beta power, was more pronounced in relapsing than in abstaining patients. Further, after 6 months of treatment, only the abstaining patients showed an increase in slow activity, a decrease in fast alpha, an acceleration of the delta/theta centroid, and a deceleration of the alpha centroid, reflecting a normalization of brain function. These findings suggest that EEG measures may serve as useful prognostic indicators in alcoholism. However, notwithstanding the proven and potential applications, electrophysiological tools are often thought to have not yet been optimized as standardized outcome measures for their use in clinical trials (Cho et al. 2005).

## Cognitive Remediation Techniques

Cognitive remediation is a neurobehavioral treatment that uses repetitive practice and compensatory and adaptive strategies to facilitate improvement in targeted cognitive areas, such as memory, attention, and problem solving (Medalia and Choi 2009). Cognitive remediation, also called “cognitive retraining” or “cognitive rehabilitation,” has been applied to several neurological and psychiatric conditions (for a review, see Langenbahn et al. 2013), including alcoholism (Allen et al. 1997; Godfrey and Knight 1985; McCrady and Smith 1986). Electrophysiological measures can serve as metrics of cognitive functioning during pre- and posttreatment of cognitive remediation. For example, Horowitz-Kraus and Breznitz (2009) found that brain activity changed in dyslexic patients as a result of working-memory training, as evidenced by an increase in both working-memory capacity and the amplitude of the ERN component of the ERPs. When ERN amplitudes increased, the percentage of errors on the Sternberg test of working memory decreased, suggesting that by expanding the working-memory capacity, larger units of information are retained in the system, enabling more effective error detection.

According to Campanella and colleagues (2011), electrophysiological methods can guide the clinician to optimize the medication regimen tailored to a patient’s cognitive profile and adopt a kind of “personalized medicine” (Campanella et al. 2011). For example, alcoholic patients with attentional biases toward alcohol cues (indexed by increased P100 to probes replacing drug cues), but with intact inhibitory processes (indexed by normal No-Go P3 component), may hypothetically benefit more from acamprosate (which regulates the increased cerebral glutamate activity by restoring the balance between excitatory and inhibitory neurotransmission), by reducing the hyperexcitability that occurs during early abstinence. However, alcoholic patients with a reversed cognitive pattern (i.e., a deficient inhibitory mechanism with altered No-Go P3 but without any attentional biases toward alcohol cues indexed by normal P100) will likely improve with naltrexone (an opioid antagonist which blocks the release of alcohol-induced dopamine), which reduces or eliminates the positive reactions associated with the urge to drink and inhibits a dominant response (drinking) by reducing the reinforcing/reward effects of alcohol (Campanella et al. 2011). Although these hypothesized applications are intriguing, more studies are needed to find empirical support for the possible role of electrophysiological measures in this type of personalized medicine; caution is suggested for any direct clinical application based on these findings and their implications, as more empirical evidence is still needed.

## Neurofeedback

The treatment of addictive disorders by EEG biofeedback (or neurofeedback, as it often is called) was first popularized by the work of Eugene Peniston (Peniston and Kulkosky 1989, 1990, 1991; Saxby and Peniston 1995) and became popularly known as the Peniston Protocol. This approach employed independent auditory feedback of two slow brain-wave frequencies, alpha (8 to 13 Hz) and theta (4 to 8 Hz) in an eyes-closed condition to produce a hypnagogic state. Patients were taught before neurofeedback to use “success imagery” (being sober, refusing offers of alcohol, living confidently, and being happy) as they drifted down into an alpha-theta state. Repeated sessions reportedly resulted in long-term abstinence and changes in personality (cf. Sokhadze et al. 2008). Several studies have reported that the Peniston neurofeedback method has been effective in achieving abstinence and improving cognitive and behavioral symptoms (cf. Saxby and Peniston 1995). For example, Saxby and Peniston (1995) reported that only 1 of 14 patients had relapsed by 21 months after neurofeedback training.

Compared with a nonalcoholic control group and a traditionally treated alcoholic control group, alcoholics who received brainwave training showed significant increases in percentages of alpha and theta rhythms in the EEG traces (as visually assessed by blind raters), increased amplitude in alpha rhythm, and sharp reductions in depression scores compared with the control groups (Peniston and Kulkosky 1989). Neurofeedback techniques have been found to be effective for treating alcohol and other SUDs (Sokhadze et al. 2008; Trudeau et al. 2009) and to improve performance and well-being in individuals with other behavioral/emotional problems (Gruzelier 2009). According to Gruzelier (2009), neuroanatomical circuitry underlying alpha-theta neurofeedback involves cognitive as well as affective/motivational functions subserved by the interaction between distal and widely distributed brain connections, mainly from the ascending mescencephalic-cortical arousal system and limbic circuits. These studies suggest that neurofeedback methods may become effective therapeutic tools for AUD, although more studies are needed to both confirm and enhance their applications.

## DBS

Another potential area for the application of electrophysiological measures in the treatment of addiction is DBS (Kuhn et al. 2011, 2013; Voges et al. 2013). DBS involves electrical stimulation of high-frequency electrodes surgically placed in one or more specific brain region(s), including the ventral intermediate nucleus of the thalamus, the subthalamic nucleus, and the internal segment of the globus pallidus. This technique is aimed at ameliorating the symptoms of movements, cognition, and emotions in several neuropsychiatric conditions (Luigjes et al. 2013; Perlmutter and Mink 2006). As a result of its successful application and approval for several neurological disorders, DBS is thought to be a powerful tool for modulating dysregulated networks and also has been considered for treating substance addiction (cf. Kuhn et al. 2013). DBS is a surgical procedure performed in the treatment/rehabilitation of some neurological conditions (Lyons 2011) and SUDs (Kuhn et al. 2013; Munte et al. 2013). Although electrophysiological measures do not have any direct role in this neurosurgical procedure, per se, they can aid in followup and maintenance of cognitive functioning in patients with DBS. For example, Kuhn and colleagues (2011) assessed cognitive control using psychometric and electrophysiological measures in severely alcohol-dependent patients who recently had undergone DBS procedures for addiction treatment and found that DBS drastically reduced addictive behavior and craving. Further, error-related negativity, an electrophysiological marker of error processing linked to anterior mid-cingulate cortex functioning, was altered after the DBS surgery, an effect that could be reversed by periods without stimulation. This case illustrates the utility of electrophysiological measures to aid in the follow-up treatment in DBS. Fins and Shapiro (2014) suggest that brain-mapping methods may advance the potential applications of DBS in the perspective of personalized medicine. However, further electrophysiological research is warranted to understand and optimize the effectiveness and outcome of this potentially promising method.

## Summary and Future Directions

This article has summarized and discussed several electrophysiological measures and tools available for alcoholism research. (See the accompanying table for a summary of findings for each method and measure.) Although advances in several electrophysiological methods are highlighted, some of these newer techniques have not yet been used to explore AUD. Nevertheless, many of these tools have potential for applications to characterize and understand alcoholism and other related disorders, and recommendations have been made to apply these novel tools or techniques to the field of alcoholism. Further, the translational potential for electrophysiological measures of brain function as endophenotypes and as valuable tools to aid in prevention, diagnosis, treatment, and rehabilitation have been briefly discussed.

Future research will focus on newer and more effective electrophysiological techniques available for neurocognitive, genetic, and clinical research. Given that electrophysiological measures hold promise as effective endophenotypes for gene discovery, these tools have potential for drug discovery as well as for a range of clinical applications. A variety of sophisticated statistical techniques (e.g., developmental trajectories), which will allow systematic research on several key electrophysiological measures, will be useful to highlight longitudinal aspects of cognitive development as well as the nature and course of the disorder under investigation. Furthermore, recent research has capitalized on the potential of using complementary information from neuroimaging methods and electrophysiological measures by performing multimodal brain imaging (Uludag and Roebroeck 2014) (e.g., combined EEG–fMRI studies), which has offered and will offer remarkable findings to understand the disorder in a better light than any single method can potentially promise (He and Liu 2008). The studies using multimodal imaging approaches are growing rapidly by implementing a simultaneous EEG–fMRI protocol aimed at achieving both high temporal and spatial resolution of human brain function (Huster et al. 2012; Mantini et al. 2010), and this approach already has been found to be highly useful in many clinical conditions (Gotman and Pittau 2011; Shafi et al. 2012), including alcoholism (De Ridder et al. 2011; Karch et al. 2008). As a final note, as advancement in technology enhances the opportunity for further applications in clinical research in all spheres, many more tools are being developed in the electrophysiological arsenal to be effectively used to address the current and future challenges.

## Figures and Tables

**Table:** Summary of Major Electrophysiological Findings in Alcoholism

**Method/Measure**	**Function/Dysfunction**	**Findings in Alcoholics**	**Findings in High-Risk (HR) Offspring/Relatives**
Resting electroencephalogram (EEG): delta power (1 to 3 Hz)	Integration of cerebral activity with homeostatic processes. Increased awake delta power is related to neurological and psychiatric conditions.	Equivocal (both increase and decrease reported).	No significant findings reported.
Resting EEG: theta power (4 to 7 Hz)	May be involved in biological rhythms and cognitive states. Increased awake theta power is related to neurological and psychiatric conditions.	Equivocal (both increase and decrease reported).	No abnormal theta power found.
Resting alpha power (8 to 12 Hz)	Higher cognitive function and brain maturation; integrative brain function.	Equivocal (both increase and decrease reported).	Equivocal (both increase and decrease reported).
Resting EEG: beta power (12 to 28 Hz)	Indicative of awake and active state. Increased beta may be related to increased neural excitability.	Increased power.	Increased power.
Resting EEG: dipole source modeling	Brain sources of scalp potentials. Abnormal source activity may be seen in clinical conditions.	No studies as yet.	No studies as yet.
Resting EEG: coherence	Functional connectivity between brain regions. Frequency-specific and region-specific coherence indicative of strength of coupling, network interaction, and brain maturation.	Increased high theta coherence; inconclusive in other frequencies.	Tenuous findings of increased coherence in several frequency bands.
EEG/event-related oscillations (ERO): graph theoretical method	Topological properties (i.e., regions and connectivity) of brain networks.	Graph theoretical indices of EEG data specific to alcoholic subjects have been elicited.	No studies as yet.
Resting EEG: microstate analysis	Possible indices of resting state networks in the brain.	No studies as yet.	No studies as yet.
EEG trilinear modeling	Estimation of a set of spatial and spectral components of brain potentials.	Significant linkage and association between trilinear component of EEG. beta band and a gamma-aminobutyric acid type A (GABA_A_) receptor gene (*GABRA2*) in Collaborative Study on the Genetics of Alcoholism (COGA) densely affected alcoholic families.	No studies as yet.
EP: auditory brainstem potentials	Integrity of sensory pathways; sensory processing.	Prolonged latencies in several auditory brainstem potential peaks.	No change in amplitude or latency.
EP: P1/P100	Basic perceptual processing of the stimulus; modulated by physical characteristics of the stimulus.	Decreased amplitudes, delayed latencies and topographic changes in visual paradigms.	No significant findings reported.
Event-related potential (ERP): N1/N100	Attentional modulation during perceptual processing of the stimulus; selective attention.	Decreased amplitude.	Decreased amplitude.
ERP: MMN	Automatic stimulus change detection; central auditory processing mechanism.	Findings are equivocal.	Findings are equivocal.
ERP: ERN/Ne	Preconscious error-detection mechanism.	Findings are equivocal.	No studies as yet.
ERP: N2/N200	Detection of response conflict (conflict monitoring); response inhibition; feedback processing.	Decreased amplitude and delayed latency.	Decreased amplitude and delayed latency.
ERP; P3/P300	Context/demand processing; stimulus significance; conscious attention; working memory.	Decreased amplitude and delayed latency.	Decreased amplitude and delayed latency.
ERP: N4/N400	Language/semantic processing; detection of incongruity in word meaning; semantic priming effects.	Decreased amplitude and delayed latency in word incongruity studies; lack of attenuation to primed words and no differentiation between primed vs. unprimed words (no priming effect).	Lack of attenuation to primed words; no differentiation between primed vs. unprimed words (no priming effect).
ERP: dipole source modeling	Brain sources of scalp potentials. Abnormal source activity may be seen in clinical conditions.	Changes in the location of brain sources for P1, N1, P2, and MMN.	No studies as yet.
ERP: current source density (CSD)	Estimation of the local radial current density and flow; spatial filtering; identification of neural sources. Changes in source activity in strength or location may suggest abnormality.	Changes in the topography and strength of activation for P3.	Changes in the topography and strength of activation for P3.
ERP: low-resolution brain electromagnetic tomography (LORETA)	Estimation of current density in voxels; identification of neural sources; patterns of activation and connectivity. Changes in current density activation level and pattern may suggest abnormality.	Changes in current density activation level and pattern for N2 and P3.	Changes in current density activation level and pattern for N2 and P3.
ERP: principal component analysis (PCA)	Decomposition of signals into orthogonal components representing distinct topographic activity patterns.	No conclusive findings.	No conclusive findings.
ERP: independent component analysis (ICA)	Decomposition of signals into a sum of temporally independent and spatially fixed components.	Changes in activation strength in ICA components for N2 and P3.	No studies as yet.
ERP: trilinear modeling	Estimation of a set of spatial and temporal components of brain potentials; simultaneous comparison of components across subjects and conditions is possible.	Significant linkage between time warped P3-related trilinear components in visual oddball paradigm in COGA densely affected alcoholic families.	No studies as yet.
ERO: delta (1 to 3.5 Hz) power	Signal detection and decision making; context/reward processing.	Decreased evoked and total delta power during P3 response window.	Decreased evoked and total delta power during P3 response window.
ERO: theta (3.5 to 7.5 Hz) power	Conscious awareness; episodic retrieval; recognition memory; executive control; inhibitory processing; working memory.	Decreased evoked and total theta power during N2 and P3 time window.	Decreased evoked and total theta power during N2 and P3 time window.
ERO: gamma (29 to 45 Hz) power	Visual perception, cognitive integrative function such as “binding”, and top-down (frontal) control during sensory processing.	Reduction in early evoked gamma power at frontal regions during target processing.	Reduction in early evoked gamma power at posterior regions during target processing.
ERO: event-related desynchronization and synchronization (ERD/ERS)	ERD represents an activated cortical area with increased excitability, while ERS indicates a deactivated cortical area with decreased excitability.	No studies as yet.	No studies as yet.
ERO: coherence	Functional interaction and connectivity across brain regions.	Increased wavelet coherence in theta (4 to 8 Hz), alpha (8 to 13 Hz) and gamma (50 to 60 Hz) bands at frontal and occipital regions during 100 to 200 ms poststimulus of target processing.	No studies as yet.
ERO: phase synchronization	Functional interactions and connectivity across brain regions; long-range neural integration.	Impaired synchronization and loss of lateralization, most prominently in alpha and lower beta frequency bands during mental rehearsal of pictures.	No studies as yet.
EEG/ERO: Granger causality	Directional influences and pathways in neural networks; couplings (connectivity) and information exchange across brain regions.	No studies as yet.	No studies as yet.

## Footnotes

2 In Go/No-Go Tasks, stimuli are presented in a continuous stream and participants perform a binary decision on each stimulus. One of the outcomes requires participants to make a motor response (Go), whereas the other requires participants to withhold a response (No-Go). Accuracy and reaction time are measured for each event. Go events typically occur with higher frequency than No-Go events.

3 In a CPT, subjects are presented with a stream of letters and must respond to one of the letters (e.g., “X”) only when it follows a specific letter (e.g., “O”) and refrain from responding to any other combination of letters (e.g., in the case of “X” preceded by any other letter of the alphabet, or any other letter following “O”, no response is made).This is called an “O-X” paradigm.

4 In stop-signal tasks, participants have to respond as quickly as possible to a particular stimulus feature (e.g. color, shape, identity, or location). On a minority of the trials, the go stimulus is followed by an additional signal (e.g. an auditory tone or a visual cue), which instructs participants to withhold their planned response.

5 EOG signals are electrical potentials that are generated from movements of the eyeballs, and are measured by pairs of electrodes typically placed above and below or to the left and right of the eye.
